# Mathematical Modelling of Molecular Pathways Enabling Tumour Cell Invasion and Migration

**DOI:** 10.1371/journal.pcbi.1004571

**Published:** 2015-11-03

**Authors:** David P. A. Cohen, Loredana Martignetti, Sylvie Robine, Emmanuel Barillot, Andrei Zinovyev, Laurence Calzone

**Affiliations:** 1 Institut Curie, Paris, France; 2 INSERM, U900, Paris, France; 3 Mines ParisTech, Fontainebleau, Paris, France; 4 CNRS UMR144, Paris, France; National Research Council of Canada, CANADA

## Abstract

Understanding the etiology of metastasis is very important in clinical perspective, since it is estimated that metastasis accounts for 90% of cancer patient mortality. Metastasis results from a sequence of multiple steps including invasion and migration. The early stages of metastasis are tightly controlled in normal cells and can be drastically affected by malignant mutations; therefore, they might constitute the principal determinants of the overall metastatic rate even if the later stages take long to occur. To elucidate the role of individual mutations or their combinations affecting the metastatic development, a logical model has been constructed that recapitulates published experimental results of known gene perturbations on local invasion and migration processes, and predict the effect of not yet experimentally assessed mutations. The model has been validated using experimental data on transcriptome dynamics following TGF-β-dependent induction of Epithelial to Mesenchymal Transition in lung cancer cell lines. A method to associate gene expression profiles with different stable state solutions of the logical model has been developed for that purpose. In addition, we have systematically predicted alleviating (masking) and synergistic pairwise genetic interactions between the genes composing the model with respect to the probability of acquiring the metastatic phenotype. We focused on several unexpected synergistic genetic interactions leading to theoretically very high metastasis probability. Among them, the synergistic combination of Notch overexpression and p53 deletion shows one of the strongest effects, which is in agreement with a recent published experiment in a mouse model of gut cancer. The mathematical model can recapitulate experimental mutations in both cell line and mouse models. Furthermore, the model predicts new gene perturbations that affect the early steps of metastasis underlying potential intervention points for innovative therapeutic strategies in oncology.

## Author Summary

We provide here a logical model that proposes gene/pathway candidates that could abrogate metastasis. The model explores the mechanisms and interplays between pathways that are involved in the process, identifies the main players in these mechanisms and gives some insight on how the pathways could be altered. The model reproduces phenotypes of published experimental results such as the double mutant Notch^+/+^/p53^-/-^. We have also developed two methods: (1) to predict genetic interactions and (2) to match transcriptomics data to the attractors of a logical model and validate the model on cell line experiments.

## Introduction

Understanding the etiology of metastasis is very important in clinical perspective. Despite the progress with treatment of the primary tumours, the chances of survival for a patient decrease tremendously once metastases have developed [1]. It is estimated that metastasis accounts for 90% of cancer patient mortality [2]. It is now understood that the metastatic process follows a sequence of multiple steps, each being characterised by a small probability of success: 1) infiltration of tumour cells into the adjacent tissue, 2) migration of tumour cells towards vessels, 3) intravasation of tumour cells by breaching through the endothelial monolayer, 4) travelling in the circulatory or in the lymphoid system, 5) extravasation when circulating tumour cells re-enter a distant tissue, and 6) colonisation and proliferation in distant organs [3]. The early stages of invasion and migration are tightly controlled in normal cells and can be drastically affected by malignant mutations. It has been shown indeed that primary and secondary tumours have a common gene signature [4] that mediates the initial stages of metastasis while extravasation and colony formation by a (tumour) cell does not require malignant gene alterations [5], supporting the idea that the later stages of metastasis are affected by the anatomical architecture of the vascular system [6].

Here, we focus on the ability of cancer cells to infiltrate and migrate into the surrounding tissue. The first step towards the formation of secondary tumours is acquiring the ability to migrate. In order to gain motile capacity, epithelial cells need to change their morphology through Epithelial to Mesenchymal Transition (EMT), a process which occurs during development (EMT type 1), wound healing (EMT type 2) and under pathological conditions such as cancer (EMT type 3) [7,8]. EMT type 3 is characterised by both loss of E-cadherin (*cdh1*) and invasive properties at the invasive front of the tumour [9]. Gene expression of E-cadherin is inhibited by the transcription factors Snai1/2, Zeb1/2 and Twist1, while gene expression of N-cadherin (*cdh2*) is induced by the same transcription factors [8,10,11]. These transcription factors activate other genes that initiate EMT [11–13] and are induced by several signalling pathways including TGF-β, NF-κB, Wnt and Notch pathways [8,14,15]. On the contrary, the transcription factor p53 has been shown to inhibit EMT via degradation of Snai2 [16]; however, a p53 loss of function (LoF) alone is not sufficient to induce EMT [17]. After the switch of E-cadherin to N-cadherin expression, the cell-cell contacts are weakened [18,19] and cancer cells can pass the basal membrane to infiltrate the surrounding tissue [20]. The process of local invasion can be active since tumour cells can secrete Matrix Metalloproteinases (MMPs) that dissolve the lamina propria [21]. MMPs are also able to digest other components of the extracellular matrix (ECM) and thereby to release growth factors and cytokines that are attached to the ECM [21,22] which in turn activate the tumour cell’s ability to propagate the dissolvement of the lamina propria. Finally, after dissolving the lamina propria and invading the (local) stroma, cancer cells can migrate to distant sites by intravasation and extravasation of the vascular system [2]. To gain insight in the regulation of the metastatic process, several groups have developed mathematical models of various aspects of it [23–29] (S1 Text).

Our aim is to understand the role of gene alterations in the development of metastasis. In many (experimental or *in silico*) models, EMT is described as a very important step in acquiring metastasis and even considered to be synonymous to appearance of metastasis [30–32]. Due to EMT role in metastasis, much research has been performed to elucidate its regulation. The regulation of EMT is known to be complex and simple intuition is not sufficient to comprehend how genetic alterations (mutations and copy number variations) affect it. Logical modelling can give qualitative insight on how they could affect EMT and subsequently metastasis.

Previously, we have constructed a detailed map of molecular interactions involved in EMT regulation which is freely available at [33], and based on its structural analysis, we hypothesized a simple qualitative mechanism of EMT induction through upregulation of Notch and simultaneously deletion of *p53*. This prediction has been experimentally validated in a mouse model of colon carcinoma [31].

In the present study, we significantly extend the biological context and provide a mathematical framework for the description of the necessary conditions for having metastasis, going beyond the regulation of EMT only. We take into consideration the gained motility and ability to invade as determinants of the metastatic process. For this purpose we largely extended and re-designed the signalling network including more molecular players and phenotypes, and translated the network into a formal mathematical model, allowing prediction of the metastasis probability and the systematic analysis of mutant properties. Therefore, this work represents a significant progress with respect to the previous results, allowing reconsideration of the qualitative hypothesis suggested before using a formal mathematical modelling approach.

First, we introduce a logical model that recapitulates the molecular biology of the early steps in metastasis. The construction of the influence network and the choice of the logical rules are both based on knowledge derived from scientific articles. The final readouts of the model are the phenotype variables *CellCycleArrest*, *Apoptosis* and the aggregated phenotype *Metastasis* that combines the phenotypes *EMT*, *Invasion* and *Migration*.

We have chosen those final read-outs, as we believe that a metastatic phenotype depends on the occurrence of EMT, invasion and migration. In addition, apoptosis is of importance to the system as during healthy conditions, the cells undergo apoptosis when the cells detach from the basal membrane [34]. Suppressing apoptosis during migration is a required key feature. Our interest in cell cycle arrest is due to results of the mouse model [31] that show decreased proliferation. We try to model this feature in our logical model by looking at the regulation of cell cycle arrest. We did not focus on other phenotypes (or cancer hallmarks) such as proliferation explicitly, senescence, or angiogenesis. These are often considered in cancer studies but they were out of the scope of this work, which focused on depicting early invasion modes and not specifically on how tumour growth is regulated. The model inputs have been selected to represent external signals necessary for the metastasis initiation pathways. The Boolean model that we show here describes a possible regulation of the metastatic potential of a single tumour cell and not of multiple cells or a tissue.

We provide a simplified version of the model where some genes are grouped into modules (or pathways) allowing an analysis based on pathways rather than individual genes. Both versions of the models are validated by reproducing the phenotypic read-outs of published experimental mouse and cell line models.

We then analyse the two models and formulate several types of predictions: at the level of individual genes, e.g. exploring the individual role of each EMT inducer in metastasis; and at the level of pathways, e.g. investigating the functional role of each pathway in triggering metastasis. The logical models can suggest a systematic mechanistic explanation for the majority of experimentally validated mutations on the local invasion and migration processes. Moreover, we were able to establish a link between the solutions of the mathematical model and the gene expression data from cell lines in which EMT was transiently induced. In addition, we have applied this method to the analysis of transcriptomes of tumour biopsies.

Lastly, we investigate how genetic interactions between different gene mutations can affect the probability of reaching a metastatic outcome. Our analysis predicts the effect of single mutations and the genetic interactions between two single mutations with respect to several cellular phenotypes. Our model proves an exceptionally efficient synergetic effect of increased activity of Notch in combination with a decreased activity of p53 on metastasis in accordance with our previous work [31].

## Materials and Methods

### The influence network

The construction of the influence network is based on scientific articles that describe the interactions between nodes of the model. We first selected the main genes or proteins that may contribute to activating EMT, regulating early invasion and triggering metastasis. We then searched for experimentally proven physical interactions that would link all these players, and simplified the detailed mechanisms into an influence network. For example, it has been shown experimentally that AKT protein phosphorylates and stabilises MDM2, which in turn inhibits p53 by forming a complex, leading to protein degradation of p53. We simplified the biochemical reactions by a negative influence from AKT to p53. The influence network is then translated into a mathematical model using Boolean formalism (see below for details). We verified the coherence of the network by comparing the outcome of the perturbed model to the observed phenotypes of mutants found in the literature. The final model is the result of several iterations that led to the accurate description of most of the published mutants related to the genes included in our model. Once the model was able to reproduce most of the published mutant experiments, we simulated mutants and conditions not yet assessed experimentally and predicted the outcome.

### The Boolean formalism

From the *influence network* recapitulating known facts about the processes, we develop a mathematical model based on the Boolean formalism. To do so, we associate to a node of the influence network a corresponding *Boolean variable*. The variables can take two values: 0 for absent or inactive (OFF), and 1 for present or active (ON). The variables change their value according to a logical rule assigned to them. The *state of a variable* will thus depend on its *logical rule*, which is based on logical statements, i.e., on a function of the node regulators linked with logical connectors AND (also denoted as &), OR (|) and NOT (!). A *state of the model* corresponds to a vector of all variable states. All possible model states are connected into a *transition graph* where the nodes are model states and the edges correspond to possible transitions from one model state to another. The transition graph is based on asynchronous update, i.e., each variable in the model state is updated one at a time as opposed to all together in the synchronous update strategy. *Attractors* of the model refer to long-term asymptotic behaviours of the system. Two types of attractors are identified: *stable states*, when the system has reached a model state whose successor in the transition graph is itself; and *cyclic attractors*, when trajectories in the transition graph lead to a group of model states that are cycling. In this model, no cyclic attractors were found for the wild type case. However, we do not guarantee the non-existence of cyclic attractors in some of the perturbed cases, as perturbations of the model may create new dynamics.

### The logical rules

A logical rule is written for each variable of the model, corresponding to a node in the influence network, in order to define how its status evolves (ON or OFF). In this rule, the variables of the input nodes are linked by logical connectors according to what is known about their combined activities. There are several cases to consider: (1) The simplest logical rule that can be assigned is when a variable depends on the activity of a single input: for instance, the transcription factor Twist induces the transcription of the *cdh2* gene (see Table 1); (2) In the case of an input that has a negative effect on the activity of its target, the Boolean operator “NOT” or “!” is used: EMT is, for example, activated by CDH2 but inactivated by CDH1, thus for EMT to be activate, CDH1 should be OFF and CDH2 should be ON. The complete logical rule for the activation of EMT will be EMT = 1 (ON) if CDH2 &! CDH1 (see Table 1); (3) In some cases, a gene can be activated by two independent genes reflecting two different conditions and thus inputs are linked by an OR, e.g., DKK can be activated either by CTNNB1 or by NICD, independently of each other; (4) In some other cases, two activators are linked by an AND connector, e.g., ZEB2 whose activity depends on several inputs including TWIST1 & SNAI2 which are needed simultaneously: it has been observed experimentally that both transcription factors Twist1 and Snai2 are required to induce gene expression of *zeb2*. All models are available in GINsim format in S1 File.

**Table 1:** Logical rules describing the activity of a node.

Node	Rule
AKT1	CTNNB1 & (NICD | TGFbeta | GF | CDH2) & ! p53 & ! miR34 & ! CDH1
AKT2	TWIST1 & (TGFbeta | GF | CDH2) & !(miR203 | miR34 | p53)
CDH1	!TWIST1 & ! SNAI2 & ! ZEB1 & ! ZEB2 & ! SNAI1 & ! AKT2
CDH2	TWIST1
CTNNB1	!DKK1 & ! p53 & ! AKT1 & ! miR34 & ! miR200 & ! CDH1 & ! CDH2 & ! p63
DKK1	CTNNB1 | NICD
ERK	(SMAD | CDH2 | GF | NICD) & ! AKT1
GF	!CDH1 & (GF | CDH2)
miR200	(p63 | p53 | p73) & !(AKT2 | SNAI1 | SNAI2 | ZEB1 | ZEB2)
miR203	p53 & !(SNAI1 | ZEB1 | ZEB2)
miR34	!(SNAI1 | ZEB1 | ZEB2) & (p53 | p73) & AKT2 & ! p63 & ! AKT1
NICD	!p53 & ! p63 & ! p73 & ! miR200 & ! miR34 & ECM
p21	((SMAD & NICD) | p63 | p53 | p73 | AKT2) & !(AKT1 | ERK)
p53	(DNAdamage | CTNNB1 | NICD | miR34) & ! SNAI2 & ! p73 & ! AKT1 & ! AKT2
p63	DNAdamage & ! NICD & ! AKT1 & ! AKT2 & ! p53 & ! miR203
p73	DNAdamage & ! p53 & ! ZEB1 & ! AKT1 & ! AKT2
SMAD	TGFbeta & ! miR200 & ! miR203
SNAI1	(NICD | TWIST1) & ! miR203 & ! miR34 & ! p53 & ! CTNNB1
SNAI2	(TWIST1 | CTNNB1 | NICD) & ! miR200 & ! p53 & ! miR203
TGFbeta	(ECM | NICD) & ! CTNNB1
TWIST1	CTNNB1 | NICD | SNAI1
VIM	CTNNB1 | ZEB2
ZEB1	((TWIST1 & SNAI1) | CTNNB1 | SNAI2 | NICD) & ! miR200
ZEB2	(SNAI1 | (SNAI2 & TWIST1) | NICD) & ! miR200 & ! miR203
CellCycleArrest	(miR203 | miR200 | miR34 | ZEB2 | p21) & ! AKT1
Apoptosis	(p53 | p63 | p73 | miR200 | miR34) & ! ZEB2 & ! AKT1 & ! ERK
EMT	CDH2 & ! CDH1
Invasion	(SMAD & CDH2) | CTNNB1
Migration	VIM & AKT2 & ERK & ! miR200 & ! AKT1 & EMT & Invasion & ! p63
Metastasis	Migration

### Computing phenotype probabilities using MaBoSS

MaBoSS is a C++ software for simulating continuous/discrete time Markov processes, defined on the state transition graph describing the dynamics of a Boolean network. The rates up (change from OFF to ON) and down (from ON to OFF) for each node are explicitly provided in the MaBoSS configuration file together with logical functions, which allows working with physical time explicitly. All rates are set to be 1 in this model since it is difficult to estimate them from available experimental facts. Probabilities to reach a phenotype are computed as the probability for the phenotype variable to have the value ON, by simulating random walks on the probabilistic state transition graph. The probabilities for the selected outputs are reported for each mutant based on predefined initial conditions (which can be all random). Since a state in the state transition graph can combine the activation of several phenotype variables, not all phenotype probabilities appear to be mutually exclusive. For example, *Apoptosis* phenotype variable activation is always accompanied by activation of *CellCycleArrest* phenotype variable (because p53 is a transcription factor of p21, responsible for cell cycle arrest, and the miRNAs, activated by the p53 and its family members, lead to a cell cycle arrest), and activation of the *Metastasis* phenotype variable is only possible when all three *EMT*, *Invasion* and *Migration* phenotype variables are activated.

With MaBoSS, we can predict an increase or decrease of a phenotype probability when the model variables are altered, which may correspond to the effect of particular mutants or drug treatments. If mutation A increases the *Apoptosis* probability when compared to the *Apoptosis* probability in wild type, we conclude that mutant A is advantageous for apoptosis. All models are available in MaBoSS format in S1 File.

### Module activity

The pathway activity (synonymously, module activity) score in a tumour sample is defined as the contribution of this sample into the first principal component computed for all samples on the set of the module target genes, as it was done in [35]. This way, we test target gene sets selected from MSigDB [36] and KEGG [37] databases together with few tens of gene sets assembled by us from external sources. The gene lists for each module is provided in S5 Table. Differential activity score of each module was estimated by t-test between metastatic and non-metastatic groups and significantly active/inactive modules were selected according to p-value <0.05 condition.

### Transcriptomics data for tumour samples

We conducted our study on the publicly available data of human colon cancer from TCGA described in [38]. By excluding rectal cancers from the original dataset, the remaining 105 tumour samples were included in our analysis, classified into two groups (‘*metastatic*’ M1 = 17 tumours and ‘*non-metastatic*’ M0 = 88 tumours) according to clinical information about metastasis appearance during cancer progression.

### Transcriptomics data for cell lines

We used gene expression data generated from A549 lung adenocarcinoma cell line that was treated with TGF-β1 ligand at eight different time points [39]. In short, gene expression was measured for three replicates at each time point using Affymetrix Human Genome U133 Plus 2.0 Array. For more information about treatment and growth protocols see [40].

### Matching transcriptomics data to logical steady states on EMT-induced cancer cell lines

We followed the following six steps to link transcriptome data to the stable states of the model (described in detail in S2 Text): (1) We first matched the genes of the model with their HUGO names. For phenotypes such as *Apoptosis*, *Migration* or *Invasion*, the genes coding for CASP9, CDC42, and MMP2 were used as biomarkers, respectively. These readouts were selected as the most representative of the process; they were chosen based on the changes of the expression of a list of candidate genes we explored throughout the experiments. (2) We averaged the genes over the 3 replicates for time point T0 (initiation of experiment with no TGF-β), for T8 (identified as the beginning of EMT), for T24 (EMT in process) and for T72 (last point). (3) Using several methods (binarization algorithms available in [41]), we identified a threshold of expression and binarized the data accordingly. Among our list of genes, only 11 of them have significant expression dynamics along the experiment: *cdh1*, *cdh2*, *ctnnb1*, *egfr*, *mapk1*, *mmp2*, *smad3*, *snai2*, *tgfb1*, *vim* and *zeb1*. The other genes were either always ON or always OFF throughout the 72 hours of experiments because the expression is either above or below the threshold we set. (4) We associated a label (phenotype) to the 9 stable states of the logical model based on the activity status of the phenotype variable. (5) The similarity matrix was computed according to the following rule: for each stable state and for each time point, if a gene is ON (= 1) or OFF (= 0) in both the vector of discretized expression data and the vector of the stable state, we set the entry in the similarity matrix to 1, otherwise, it is set to 0. (6) For each time point and each stable state, we then summed up the corresponding similarity matrix row, and set an expression-based phenotypic (EBP) score for each stable state. The highest EBP score for each time point corresponds to the phenotype that is the closest to the studied sample and is representative of the status of the cells.

### Non-linear principal component analysis by elastic maps method

The non-linear principal manifold was constructed for the distribution of all single and double mutants of the model in the space of computed model phenotype probabilities, using elastic maps method and ViDaExpert software [42–44]. We preferred using a non-linear version of principal component analysis (PCA) for data visualisation in this case, because it is known to better preserve the local neighbourhood distance relations and allows more informative visual estimation of clusters compared to the linear PCA of the same dimension [42]. For data analysis, only those “mixed” phenotypes were selected whose probability expectation over the whole set of single and double mutants was more than 1%. It resulted in a set of 1059 single and double mutants embedded into 6-dimensional space of phenotype probabilities for which the principal manifold was computed.

### Synthetic interactions with respect to metastatic phenotype

The results of double mutants were used to quantify the level of epistasis between two model gene defects (resulting either from gain-of-function mutation of a gene or from its knock-out or loss-of-function mutation) with respect to metastatic phenotype. The level of epistasis was quantified using the simplest multiplicative null model applied for the event of *not having* metastasis: ε = (1-p_12_)-(1-p_1_)(1-p_2_), where p_1_ and p_2_ are the probabilities of having metastasis in single mutants, and p_12_ was the probability of having metastasis in the double mutant. Therefore, negative values of the epistasis score E correspond to synergistic interactions when two gene defects amplify each other’s effect stronger than expected in the multiplicative model. On the contrary, positive values correspond to alleviating effect, when the effect of one gene defect could be masked (sometimes, even reduced to zero) by the second mutation. For genetic network visualisation, we kept the most significant interactions with ε<-0.2 or ε>0.3 values. These thresholds were chosen because at these levels we observed gaps in the distribution of ε values. The complete list of interactions together with p_1_, p_2_, p_12_ and ε values can be found as a Cytoscape 3 session (S2 File).

## Results

### Construction of an influence network regulating EMT, invasion and cell migration

Mesenchymal cells are characterised by their increased motility, loss of *cdh1* (coding for E-cadherin) expression, increased expression of *cdh2* (coding for N-cadherin), and presence of *vimentin* (Vim) [7,10,45]. The EMT program can be initiated by the transcription factors *snai1*, *snai2*, *zeb1*, *zeb2* and *twist1*. They are considered to be the core regulators of EMT as each has been shown to down-regulate *cdh1* [46–50]. In turn, the genes coding for these core EMT-regulators are subjected to regulation by other signalling pathways. The TGF-β pathway has been reported to be able to induce EMT [7,51], but other pathways are also involved in EMT including Wnt, Notch and PI3K-AKT pathways [52–56].

Furthermore, microRNAs regulate the Snai and Zeb family members. For example, miR200 targets *snai2*, *zeb1* and *zeb2* mRNA [57–59] whereas miR203 targets *snai1* and *zeb2* mRNA [59], and miR34 targets *snai1* mRNA [60]. The transcription of these microRNAs is under the control of p53 [61–64]. The miR200 expression can also be induced by p63 and p73 proteins, while miR34 is only induced by p73 but is down-regulated by p63 [65–67]. The microRNAs can be down-regulated by the EMT-inducers Snai1/2, and Zeb1/2 [59,60,68]. Note that the proteins p63 and p73 have been identified as members of the p53 protein family since their amino acid sequences share high similarity with that of p53 [69]. They are able to bind to the promoters of the majority of the p53-target genes and therefore have overlapping functions in cell cycle arrest and apoptosis [70,71]. The p53-family members are involved in cross-talks with Notch and AKT pathways: p63 protein is inhibited by the Notch pathway, p53 by AKT1 and AKT2 [69,72–76] while p73 is down-regulated by p53 (itself negatively regulated by p73), AKT1, AKT2, and Zeb1 [69,72,77].

**Fig 1:**
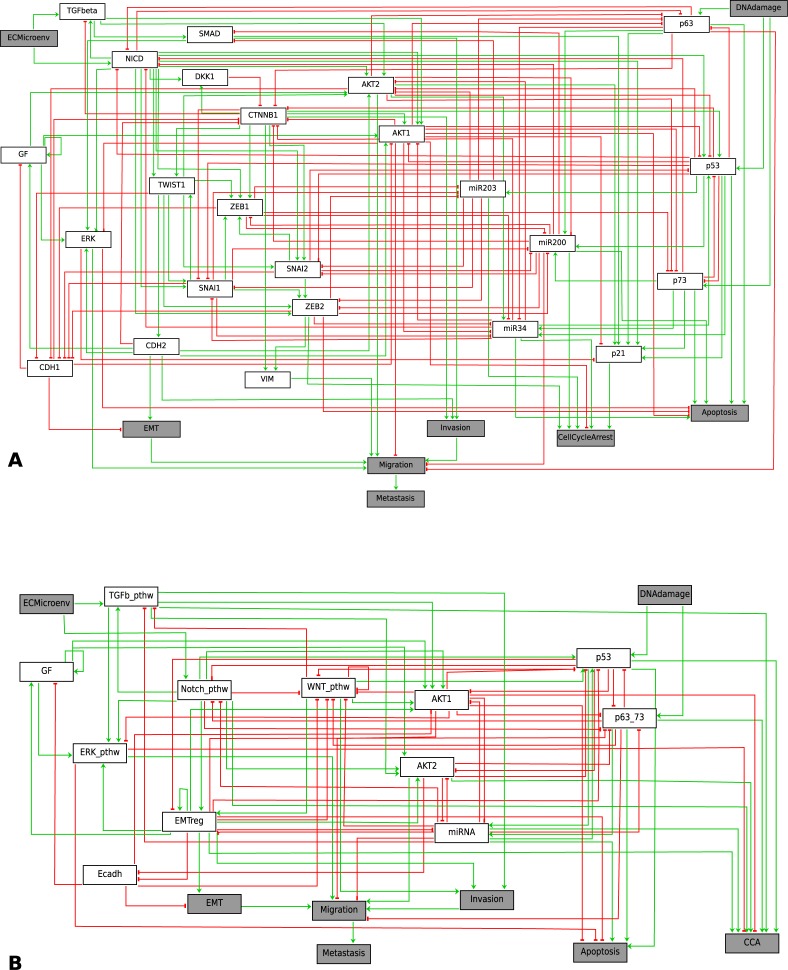
Regulatory networks of mechanisms leading to EMT, invasion, migration and metastasis. A. Detailed network of the pathways involved in metastasis. B. Modular network derived from network in A.

The PI3K-AKT pathway has been considered to be important in evading apoptosis and cell cycle arrest by modulating the TRAIL pathway, down-regulating pro-apoptotic genes and phosphorylating p21 [78–80]. More recently, AKT has been assigned additional but important roles in the development of metastasis. AKT1 suppresses apoptosis upon cell detachment (anoikis) of the ECM [34]. The different isoforms of AKT seem to have opposing roles in the regulation of microRNAs: AKT1 inhibits miR34 and activates miR200 while AKT2 inhibits miR200 and activates miR34 [81]. Another opposing role for both AKT isoforms has been found in migration. AKT1 inhibits migration by phosphorylating the protein Palladin; phosphorylated Palladin forms actin bundles that inhibit migration. AKT2 increases the protein Palladin stability and upregulates β1-integrins stimulating migration [82,83] or by inhibiting TSC2 that, in turn, activates RHO [84]. Furthermore, AKT1 inhibits cell cycle arrest while AKT2 activates it [85,86] (all these effects are shown implicitly in Fig 1A).

Extracellular stimuli are also included in the logical model. Growth factors (GF) are soluble ligands that can be excreted locally or from longer distances and are able to activate the PI3K-AKT, and MAPK pathways [87,88]. Another extracellular stimulus might be the extracellular microenvironment (ECMicroenv) with components that are not soluble including the extracellular matrix. The ligands for the TGF-β pathway can be imbedded in the extracellular matrix [89–91] and the ligands for the Notch pathway are transmembrane proteins from adjacent neighbouring cells [92,93].

These mechanisms are depicted in an influence network (Fig 1A). The network is composed of nodes and edges, where some nodes represent biochemical species (proteins, miRNAs, processes, etc.) and others represent phenotypes, and edges represent activating (green) or inhibitory (red) influences of one node onto other node. Each edge is annotated and supported by experimental papers (see S1 Table). Throughout the article, we will use the general term “phenotypes” to refer to “phenotype variables”, which correspond to the four outputs: *CellCycleArrest*, *Apoptosis*, *Metastasis* (depending on *EMT*, *Migration* and *Invasion*), and Homeostatic State (HS) as presented below.

### Mathematical modelling of the influence network

#### Construction of a logical model and its stable states

The network of Fig 1A is translated into a logical model using GINsim software [94]: a logical rule is assigned to each node of the network (Table 1, and Materials and Methods). Once the logical rules are set for each node of the network, the Boolean model can simulate solutions or outcomes that correspond to attractors in the state transition graph (see Materials and Methods for details). The model, for the wild type condition (i.e. no mutations or gene alterations), counts nine stable states for all combinations of inputs (Table 2). To each stable state, a phenotype is assigned based on the genes that are activated (variable is ON, thus equal to 1). The phenotypes identified are: *CellCycleArrest* together with *Apoptosis*; *CellCycleArrest* together with *EMT*; *Metastasis* (depending on three other processes: *EMT*, *Migration* and *Invasion*); and a stable state with only Cdh1 ON. This state corresponds to a state where metastasis is inhibited by Cdh1 activity. We refer to it as the homeostatic state (HS). It is a particular state of an epithelial cell that is not explicitly represented as a phenotype variable in this mathematical model. Four out of the nine stable states lead to *Apoptosis*, in the presence of DNA damage and absence of growth factors (GF). Two stable states show an *EMT* phenotype alone (without inducing *Metastasis*). In these stable states, *Invasion* and *Migration* are not activated because TGF-β pathway is not initially ON. The last two stable states lead to *Metastasis* in the presence of growth factors. GF activates the ERK pathway that switches off the p53-family targets and permits the triggering of events leading to metastases. Indeed, several studies have shown the importance of ERK in migration [95–97].

**Table 2:** The nine stable states of the mathematical model. The label of the columns corresponds to the phenotypic outputs.

	HS	Apop1	Apop2	Apop3	Apop4	EMT1	EMT2	M1	M2
Metastasis	0	0	0	0	0	0	0	1	1
Migration	0	0	0	0	0	0	0	1	1
Invasion	0	0	0	0	0	0	0	1	1
EMT	0	0	0	0	0	1	1	1	1
Apoptosis	0	1	1	1	1	0	0	0	0
CellCycleArrest	0	1	1	1	1	1	1	1	1
ECMicroenv	0	0	0	1	1	0	0	1	1
DNAdamage	0	1	1	1	1	1	0	1	0
GF	0	0	0	0	0	1	1	1	1
TGFbeta	0	0	0	1	1	0	0	1	1
p21	0	1	1	1	1	0	0	0	0
CDH1	1	1	1	1	1	0	0	0	0
CDH2	0	0	0	0	0	1	1	1	1
VIM	0	0	0	0	0	1	1	1	1
TWIST1	0	0	0	0	0	1	1	1	1
SNAI1	0	0	0	0	0	1	1	1	1
SNAI2	0	0	0	0	0	1	1	1	1
ZEB1	0	0	0	0	0	1	1	1	1
ZEB2	0	0	0	0	0	1	1	1	1
AKT1	0	0	0	0	0	0	0	0	0
DKK1	0	0	0	0	0	0	0	1	1
CTNNB1	0	0	0	0	0	0	0	0	0
NICD	0	0	0	0	0	0	0	1	1
p63	0	0	1	0	1	0	0	0	0
p53	0	1	0	1	0	0	0	0	0
p73	0	0	1	0	1	0	0	0	0
miR200	0	1	1	1	1	0	0	0	0
miR203	0	1	0	1	0	0	0	0	0
miR34	0	0	0	0	0	0	0	0	0
AKT2	0	0	0	0	0	1	1	1	1
ERK	0	0	0	0	0	1	1	1	1
SMAD	0	0	0	0	0	0	0	1	1

#### Testing robustness of the model with respect to small changes in the logical rules

We systematically checked the effect of changing the logical operators of the model from “OR” to “AND”, and vice versa, onto the resulting model phenotype probabilities. More specifically, we generated model variants with one change of a logical operator in one logical rule, two changes in the same logical rule, or one single change in two different logical rules, leaving the rest of logical operators the same as in the wild type model. Therefore, we considered all model variants different from the wild type model by at most two different logical operators. The analysis resulted in 8001 model variants.

We first show that the distributions of phenotype probabilities after these changes are concentrated around the wild type probability values (S6 Fig).

*Metastasis* appeared to be the least robust model phenotype, which confirms the fact that there are some necessary conditions that need to be met to lead to metastasis (illustrated by AND operators in the logical rules). If approximately 49% of changes in the logical rules have minor or no effect onto the *Metastasis* phenotype probability, some modifications in some rules changed the *Metastasis* phenotype to zero (implicating p63, p73, AKT1 variables of the model and, to a lesser extent, CTNNB1, miR34, p53). Most of the rules that concern these variables are indeed more stringent. A change from an AND gate to an OR gate for the case of p63 or AKT1 has an important impact on the metastasis process. For instance, if p63 is more present, because it is inactivated with fewer constraints, it can block more easily migration and thus, metastasis. These logical rules should be considered more carefully than the others because a mistake in defining these rules can have more drastic effects on the model properties than any other modifications.

We also performed a reproducibility analysis of individual logical stable states in the models differing from the wild type model by one or two changes in the same logical rule or one change in two logical rules as presented above. The wild type model is characterized by nine stable states, including the homeostatic state (HS) (see Table 2). 8001 model variants mentioned above are characterized by 68726 stable states counted in total. Hence, in average, each model variant is characterized by 8 or 9 stable states, which might be different from the wild type model. In total, we have counted 1176 distinct stable states in all the 8001 model variants, observed with different frequencies (S6 Table). The nine stable states of the wild type model are robustly reproducible, being the most frequently observed stable states, and accounting for 59% of all observed stable states in different model variants. Another 13% of observed stable states differ from one of the wild type stable states by only one change in the Boolean variable values (DIST_TO_WT = 1). Some modifications of logical rules for CTNNB1 or NICD lead to very rarely observed atypical but very different from the wild type stable states (DIST_TO_WT = 12). Based on all these analyses, we conclude that the nine wild type model stable states are robust and “typical” with respect to moderate random modifications of the logical rules and fragile to few targeted modifications.

#### Model reduction into a modular network

To make our modelling more insightful, we reduced the complexity by lumping variables into modules corresponding to signalling pathways: the TGF-β pathway (TGFb_pthw consisting of TGFbeta, SMAD), Notch pathway (Notch_pthw, includes activated Notch intracellular domain (NICD), the WNT pathway (WNT_pthw consisting of DKK1, CTNNB1), the p53 pathway (p53, consisting of p53), the p63-p73 proteins (p63_73 consisting of p63 and p73), the miRNA (miR34, miR200, miR203), the EMT regulators (EMT_reg including Twist1, Zeb1, Zeb2, Snai1, Snai2, Cdh2, Vim), E-cadherin (Ecadh with Cdh1), growth factors (GF), the ERK pathway (ERK_pthw: ERK), p21 is included in the *CellCycleArrest* phenotype, AKT1 module and AKT2 module. In the reduced model (Fig 1B), the inputs (ECMicroenv and DNAdamage) and (final and intermediate) outputs (*Migration*, *Invasion*, *Metastasis*, and *Apoptosi*s) are conserved. The reduced model produces the same stable states (for the wild type conditions) as those of the initial model (Fig 1A, see S4 Text).

#### Validation of the Boolean model

We simulated the genetic perturbations that correspond to published experimental settings and verified that the stable states of the mathematical model correspond to the experimental observations. An overexpression or gain of function (GoF) of a gene is simulated by forcing the value of the node to ON and a deletion or loss of function (LoF) by forcing the value of the node to OFF. We first simulated not only previously described mutants but also mutants that have not yet been experimentally validated (see S4 Table). The mathematical model is able to reproduce the experimental results of almost all described mutants. In few cases, there is a discrepancy between the mathematical and the biological model due to three reasons described below: 1) Metastasis in our logical model is defined as colonisation of tumour cells into distant organs through migration in the systemic and/or lymphatic vessels. A limitation of the cell line model is that a metastatic output cannot be measured. 2) Dosage-dependent effects cannot be modelled using the logical formalism. For example, our model predicts metastasis in a *kras* GoF while the mouse model does not develop distant metastatic tumours. A possibility for the difference is that in the mouse model the wild type *kras* allele is still present (a heterozygous mutation) while in our model KRAS mutant is homozygous. It has been reported that the remaining wild type *kras* gene has still tumour suppressive properties: it can reduce tumourigenesis in lung [98,99] and in colon cancer cell line by inhibiting proliferation [100]. Other studies in cancer cell lines that are heterozygous for *kras* mutation showed that the wild type *kras* gene in those cell lines decreased the migration and colonisation capacity [101,102] suggesting a dose-dependent effect [103]. This might indicate that mouse mutants homozygous for *kras* mutation may develop distant metastasis as predicted by our mathematical model. 3) Simulating mutations of genes that are not explicitly represented as a node in the model has its limitations because the network does not describe exactly the function of such node. For example, even though PTEN is not a variable of our model, we simulated a *pten* mutation to understand the controversial results of such deletion on metastasis in experimental models. The *pten* LoF mutations have been associated with many different types of cancers [104–106] and recently it has been demonstrated that *pten* mutations cause genomic instability [107,108]. In our model, in order to simulate a PTEN LoF, its two targets, AKT1 and AKT2, are forced to be ON: PTEN inhibits activation of AKT isoforms [109–111]. The model predicts that a PTEN LoF alone or in combination with gene mutations will reach the stable states without having metastasis while metastasis is observed in the mouse model. In our model, due to activation of AKT1 by the PTEN LoF, metastasis is prevented, because AKT1 inhibits migration as mentioned before. A recent study indicates that in PTEN-deficient tumours, AKT2 is the active isoform [112] but not AKT1. The model confirms this study: when we simulate the single AKT2 activation as a result of PTEN LoF, the model predicts a stable state in which metastasis can be reached (All references and model results are available in S4 Table).

### Role of different pathways/modules in triggering metastasis

To assess the importance of each pathway on metastasis, apoptosis and cell cycle arrest, we simulated a gain of function or a loss of function, in the reduced model, for each module and for all combinations of inputs. These simulations mean that when an important entity in a pathway is altered, it affects the whole pathway activity. The model shows that mutations leading to either GoF or LoF of each pathway have opposing results in the occurrence of migration and for the occurrence of metastasis (S2 Table). The Notch_pthw is an exception in this: both a GoF and LoF of the Notch pathway can lead to a stable state solution with metastasis ON. This might indicate that Notch (pathway) activity must be in a certain range in order to have a non-pathological effect or that Notch is important for the functioning of some dynamic feedback controls preventing metastasis (so fixing it at a particular value would destroy these feedbacks). In addition, GoF of the Notch_pthw, TGFb_pthw, ERK_pthw, EMT_reg or AKT2 shows their inhibitory role in the apoptotic process as it has been demonstrated before [113–117]. For the p53, TGF-β, EMT_reg and miRNA pathways, mutations leading to activation or inhibition have opposing results in regulating invasion when either the pathway is activated or inhibited. This effect on invasion is a direct result of having an activating or inhibiting role on EMT except for the TGF-β pathway.

The role of TGF-β pathway has been investigated. The activation of TGF-β pathway might be dependent on the micro-environment as its ligands can be found in the extracellular matrix [89–91]. The triple mutant: Notch_pthw GoF, p53 LoF and TGFb_pthw LoF leads to one stable state in which the EMT_reg is ON but no metastasis, migration, invasion or apoptosis are reachable (S2 Table) indicating that activation of TGF-β pathway (e.g., by the peripheral tumour cells more exposed to the micro-environment) is required to have metastasis in the double mutant by activating invasion [118,119].

#### Comparing the Boolean model with dynamical transcriptomic data on EMT induction and tumoral transcriptomes

In this section, the aim is to investigate if the model can predict temporal trends in the dynamics of high-throughput data in cancer cell lines or to retrospectively predict a possible appearance of metastasis using the model. Is it possible to correlate experimental or clinical data to the stable states of the model?

We first analysed the publicly available colon gene expression dataset generated by The Cancer Genome Atlas (TCGA) project [38]. Student t-test between metastatic and non-metastatic tumours was performed for genes included in the influence network to identify significant changes in their expression between the two groups (S1 Fig). Few significant differences were observed in the expression of the influence network genes in these two groups. Moreover, there was no significant differential expression of the EMT regulators observed between the two groups: expression of the EMT regulators seems to be OFF in these tumours. Since single gene-based analysis of colon cancer did not show significant differential changes in the expression of the influence network genes, we investigated the expression of the downstream targets for the transcription factors in the modular network (Notch_pthw, p53, p63_p73, EMT regulators, etc.) and recapitulated their expression into a pathway activity score (see Methods). The assumption was that the differential activity of a given transcription factor can be better reflected by a score based on the expression of its target genes rather than from its own individual expression. For the nodes that are not transcription factors (AKT1, AKT2, etc.) we considered all genes involved in the same network module. We observed that the targets of Notch pathway, Wnt pathway, p63_p73, and AKT1 and AKT2 downstream genes showed significantly higher activity score in metastatic compared to non-metastatic samples whereas p53 and microRNAs targets were less active in metastatic samples. However, the EMT regulator module showed almost no difference in module activity even if all regulators were combined in one module (See S2 and S3 Figs). Indeed, in the recent colorectal tumour-specific EMT signature established by Tan *et al*. [120] none of the genes of our EMT module were included. This means that at least in the colorectal cancer, the transcriptional dynamics of the EMT genes has relatively small amplitude, when measured on the bulk of the tumour.

Based on our analysis, we hypothesize that only a small portion of the tumour cells in a tumour sample are undergoing EMT and as a result, the EMT signal is strongly diluted when looking at the whole cell population in a sample taken from the tumour bulk. This low signal to noise ratio is not favourable to study the dynamics of the EMT process, and subsequently, the metastatic process.

We thus analysed publicly available transcriptomics data from cancer cell lines in which EMT has been induced. In a study conducted by Sartor and colleagues [39], lung carcinoma cell lines were administered with increasing amount of TGF-β and genome-wide transcriptome was measured at eight different time points, following the induction of EMT. The induction of EMT was accompanied by increasing expression for some of the EMT regulators (S4 Fig).

The expression of these regulators follows a sigmoid curve in response to TGF-β induction. For a given time-point, we checked if the expression level of the components of our model could be associated to a particular steady state of the model. We expect our model to reflect the behaviour of EMT expression level at early or late time points.

We then determined the consistency of the EMT induction experiment with the logical model following the steps presented in Materials and Methods section (and in S2 Text for details of each step). The resulting EBP (expression-based phenotypic) score of the method represents how similar an experimental condition is to a stable state. Thus, the higher the EBP score for a stable state is, the more similar the data are to that stable state, and as an extension, to the phenotype variable associated to that stable state. The computed EBP scores at each time point illustrate the evolution of the data in terms of the stable states. At T0, the highest EBP score is associated with apoptosis. At T8, both metastasis and apoptosis stable states have the highest EBP score illustrating the balance of phenotypes observed in the gradual entry into EMT. At T24 and at T72, the metastatic phenotype has the highest EBP score suggesting that EMT has occurred. Based on the above-mentioned results, the logical model is in accordance with the time course experiments in EMT-induced cell lines.

With this similarity EBP score, we have developed a method to characterise tumours in terms of a particular biological process (how the metastatic process follows EMT, migration and invasion here) with respect to the solutions of a logical model.

### Role of individual EMT regulators in triggering metastasis

To identify for each EMT regulator (Snai1, Snai2, Zeb1, Zeb2, Twist1) their specific role in the different cell fates considered in our model, we simulated LoF and GoF mutants and observed that all GoF, except for that of Snai2, led to the loss of apoptosis (S3 Table). *Metastasis* can be reached for all GoF mutants but other phenotypes can still be reached depending on the combinations of inputs. The single deletions of each EMT regulator show that Zeb2 and Twist1 are required for metastasis. Zeb2 controls migration mainly through VIM but has no direct impact on invasion. Twist1 LoF, on the contrary, modulates negatively the possibility to reach not only the metastatic phenotype but also EMT, migration and invasion. Twist1 controls EMT through Cdh2 that controls migration and EMT. Other factors, such as CTNNB1 (β-catenin) or TGF-β, play a role in triggering the metastatic process by modulating invasion or migration, but our model suggests that the main EMT regulators are Zeb2, Twist1 or Snai2, either as loss of function for Zeb2 and Twist1, or gain of function for Snai2. Note that by definition, Cdh2 is absolutely required for metastasis to occur because of its direct role in controlling EMT and migration. In our model, Cdh1 inhibits EMT (directly) and migration (through CTNNB1 and VIM) but not invasion. Since all three phenotypes are required for metastasis, the process is thus impaired when Cdh1 is over-expressed [121,122].

### Modelling synthetic interactions between genes composing the model

**Fig 2:**
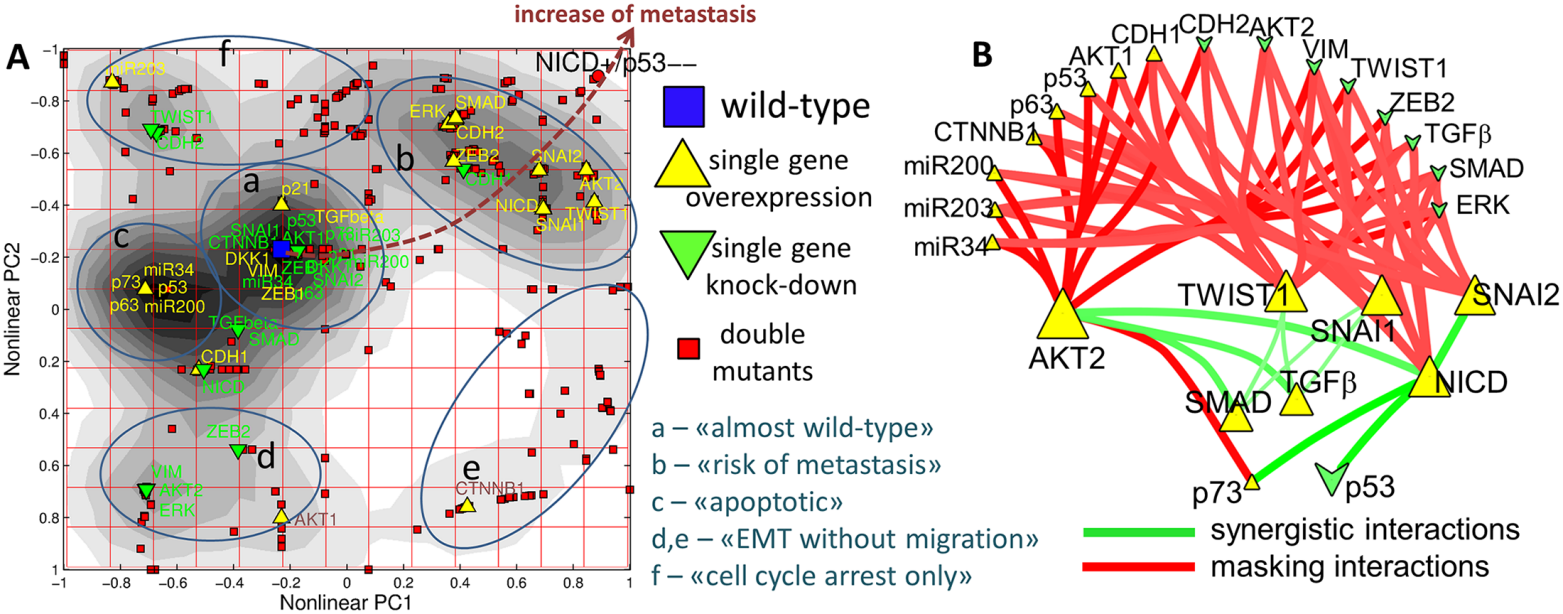
A. Genetic interactions between two mutants leading to the masking or the antagonism of a phenotype (metastasis). Application of non-linear dimension reduction for visualising the distribution of phenotype probabilities, computed with MaBoSS for all single and double mutants of the model. The grading in the background shows the density of points (mutants) projections. Six clusters are distinguished based on this grading. Wild-type model, all single over-expression and knockout mutants and the NICD GoF / p53 LoF mutant are labelled. Note that each gene pair in this plot is represented by four different double mutants (small red points) corresponding to LoF/LoF, LoF/GoF, GoF/LoF, GoF/GoF combinations. B. Genetic interaction network showing the most significant synergistic (shown in green) and alleviating (masking, showing in red) interactions between GoF and LoF mutants with respect to the probability of having metastasis. The size of the node reflects the metastasis probability for individual mutation. The thickness of the edge reflects the absolute value of epistasis measure (see Methods).

The probability of achieving the metastatic phenotype for all possible single and double mutants was systematically computed using MaBoSS [123]. Each single and double mutant is characterised by the distribution of phenotype probabilities. A non-linear PCA analysis was performed as described in Methods, which allowed to group together single and double mutants having similar effect on the model phenotypes (Fig 2A). In this plot, one can distinguish six major clusters (a to f) which can be tentatively annotated as “almost wild-type” (no significant changes in the phenotype probabilities compared to the wild-type model), “risk of metastasis” (elevated probability of having metastasis though not equal to 1), “apoptotic” (for these mutants *Apopotosis* and *CellCycleArrest* phenotypes are activated), “EMT without migration” (for these mutants, presented as two clusters, the formation of metastases cannot be accomplished because the cells did not acquire ability to migrate), “cell cycle arrest only” (these mutants are found arrested without starting EMT or invasion or apoptotic programs). The direction of increased metastasis probability is shown by dashed line in Fig 2A, which ends at NICD GoF/p53LoF double mutant for which the probability of having metastasis equals to 1, according to the model (whereas single p53 LoF mutation belongs to “almost wild type” and single NICD GoF mutation belongs to “risk of metastasis” clusters respectively).

#### Synthetic interactions with respect to metastatic phenotype

The most significant genetic interactions with respect to probability of having metastasis (see Methods) are shown in Fig 2B. The following observations can be made: (1) Hubs in this genetic interaction network are the genes for which a single mutation (GoF) leads to a significant increase in having the metastatic phenotype. These genes are *akt2*, *twist1*, *snai1*, and *snai2*; (2) There are a number of genes whose LoF or GoF lead to a significantly masking effect on the phenotype caused by the hub-gene mutations (red edges in Fig 2B). For example, overexpression of *p53* gene or knockout of *erk* gene drastically decreases the probability of metastatic phenotype in SNAI1 LoF mutant; (3) There are relatively few synergistic effects observed between single mutants (green edges in Fig 2B). Some of them have been experimentally performed while other synergistic interactions are rather unexpected such as GoF for both AKT2 and NICD, and can be a subject of further experimental work.

There are four synergistic interactions, which result in augmenting the probability of having metastasis to 100%. First, two of them are combinations of NICD GoF and p53 LoF (NICD^+^/p53^-^), or simultaneous NICD GoF and p73 GoF (NICD^+^/p73^+^). These two interactions can be considered as being dependent, since overexpression of p73 leads to downregulation of p53 function [124,125]. The other two interactions (SNAI2 GoF and NICD GoF, AKT2 GoF and NICD GoF) are potential amplifier mechanisms for appearance of metastasis in NICD GoF mutant alone.

In addition, we classified all gene pairs into five large clusters according to four different combinations of *in silico* mutation types (LoF/LoF, LoF/GoF, GoF/LoF, GoF/GoF). Inside each cluster, the gene pairs can be ranked according to the strength of the activating effect of one of the mutation combinations on the *Metastasis* phenotype (S5 Fig). Moreover, all gene pairs can be ranked according to the amplitude, i.e. the difference between the maximal and minimal metastatic phenotype probabilities among four values (LoF/LoF, LoF/GoF, GoF/LoF, GoF/GoF). The most distinguished gene pair in this analysis is p53/NICD, which is a unique and extreme case of the gene pair cluster when any combination of mutation types besides LoF/GoF makes the metastatic phenotype non-reachable (zero or close to zero probability) while the synthetic-dosage interaction LoF/GoF makes the metastatic phenotype unavoidable (probability one) (cluster 3 in S5 Fig).

#### Synthetic dosage interaction between Notch and p53 genes

**Fig 3:**
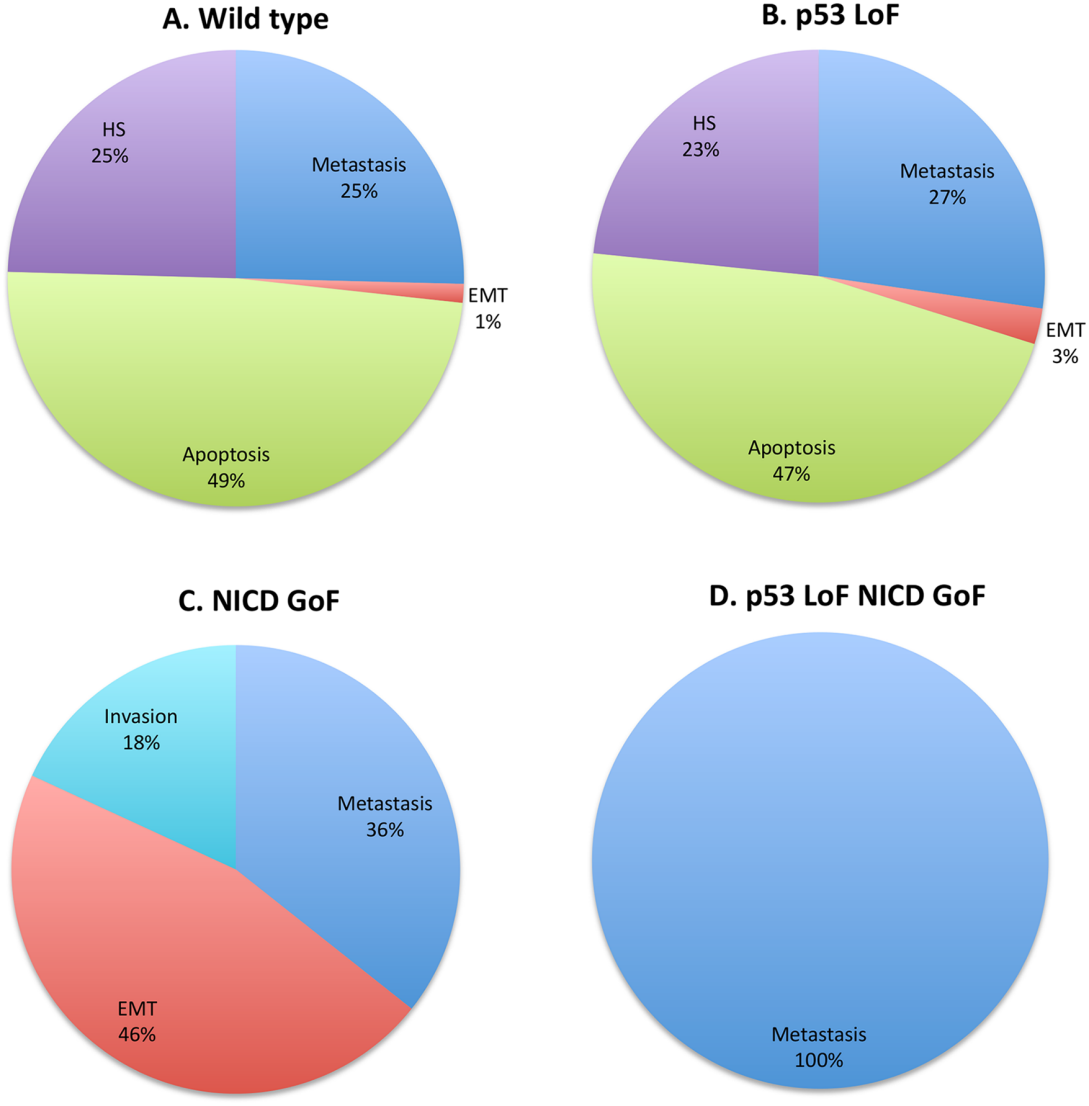
MaBoSS simulation of wild type, of individual mutations of p53 and NICD and of the double mutant. The probabilities associated with each phenotype represent the number of stochastic simulations leading to each phenotype from pre-defined initial conditions. A. Wild type, see text. B. p53 LoF, same phenotypes found in (A) are reachable but with different probabilities than wild type conditions. C. NICD GoF, apoptosis is no longer reachable. D. p53 LoF and NICD GoF, only metastasis is observed. Note that HS stands for “Homeostatic State” and CCA for “CellCycleArrest.”

Using MaBoSS, we have been able to quantify the changes of probabilities for reaching each phenotype relative to the wild type model. We are thus interested in results such as: “more or less apoptosis than in wild type”. We simulated three mutants with MaBoSS: p53 LoF (Fig 3B), NICD GoF (Fig 3C) and the double mutant NICD^+^/p53^-^ (Fig 3D). These simulations are of particular interest since they show an example of genetic interaction predicted to have the probability of metastasis phenotype equals to 1, as presented above.

The probabilities of the four phenotypes for wild type conditions are shown in Fig 3A. They show all possible phenotypes for all input configurations. Note that *Metastasis* can be only reached in the wild type for a particular set of initial conditions (ECMicroenv, GF and TGFbeta all ON), which might correspond to extreme situations. The mutant p53 LoF is very similar to the wild type in terms of possible phenotypes (Fig 2A). The NICD GoF mutation, compared to the wild type, showed an increased probability for EMT as previously reported [126]. *Metastasis* could be reached as well in this single mutant with a higher probability than in the wild type; *Apoptosis* is no longer reachable, confirming that Notch pathway is a pro-survival pathway (Figs 2A and 3C). In addition, in this mutant, *Metastasis* was clearly blocked by p53 since a loss of function of p53 in a NICD GoF mutant completely suppressed both the *EMT* and *Invasion* phenotypes present in the single mutant, and only *Metastasis* could be reached (Fig 3D). The deletion of both p63 and p73 in a NICD GOF mutant maintained the *EMT* phenotype (not shown) proving the importance of p53 in protecting the cells from metastasis.

In this context, we further investigated the role of TGF-β pathway in metastasis. Although the NICD GoF/p53 LoF double mutation has been predicted to be the best mutation to acquire metastasis, an important role for the TGF-β pathway is suggested by the model. The triple mutant NICD GoF, p53 LoF and TGF-β LoF (S4 Table) seems to suppress the metastatic process in the model: cells are able to go through EMT but cannot invade the tissue. Suppressing the TGF-β pathway might be an interesting therapeutic option to control metastasis in patients; however more studies are required to test this hypothesis.

## Discussion

In this study, we propose a logical model focusing on the specific conditions that could allow the occurrence of metastasis. Our model of the metastatic process represents its early steps: EMT, invasion and migration. A cell acquires the capability to migrate when both EMT and invasion abilities have been acquired. These steps are regulated by several signalling pathways, where genetic aberrations could influence the efficiency of metastatic process. Both the influence network and the assignment of logical rules for each node of this network have been derived from what has been published from experimental works as of today. With this model, we were able to explore known conditions (and predict new ones) required for the occurrence of metastasis. Our influence network describes the regulation of EMT, invasion, migration, cell cycle arrest and apoptosis known from the literature. In this regulatory network, cell cycle arrest and apoptosis are mechanisms or phenotypes that maintain homeostasis of organs [127] or ways to evade metastasis. Cell migration depends on pathways involving AKT, ERK, Vimentin, miR200 and p63 but also on the acquisition of EMT and invasive abilities such as producing MMPs to dissolve the *laminae propria* enabling migration to distant sites. Cells that have only invasive properties are not able to move as they are still well attached to their surrounding neighbouring cells resulting in absence of cell migration. Only when those two requirements are met and the other pathways allow migration, can metastasis occur.

The role of each EMT regulator, for acquiring invasive properties, has been investigated and the model shows that each individual EMT regulator is sufficient to induce EMT when over-expressed and with the appropriate initial conditions. The model also predicts that a LoF mutation of the EMT regulators does not affect metastasis except for ZEB2 and TWIST1: ZEB2 inhibition leads to abrogation of migration, while a TWIST1 LoF leads to inhibition of EMT, since TWIST1 is the only transcription factor that can induce transcription of *cdh2* gene which is required to have EMT. These regulators are interesting targets for therapy since both are more downstream in the metastasis’ cascade knowing that most activating mutations occur relatively more upstream e.g. KRAS and EGFR mutations.

The model has been validated using experimental data by matching the transcriptomic data with stable state solutions of the logical model. The direct comparison between stable states and gene expression of tumour samples shows no conclusive results. This may be due to that only at the front of tumours, cells undergo EMT and this signal is obscured by the bulk of the tumour [30,128]. On the other hand, the model matches well the transcriptomic data from a time course experiment of lung carcinoma cell lines in which EMT was induced by increasing concentration of TGF-β.

Qualitative simulations of the model using MaBoSS confirmed that single mutations are not sufficient to enable metastasis. Therefore, we systematically computed the level of epistasis of each two-gene mutation with respect to reaching the metastatic phenotype. We determined which double mutations are the most efficient for inducing metastasis with NICD GoF/p53 LoF mutations being the most efficient combination of gene knock-out and over-dosage, as this double mutant leads *in silico* to 100% probability of having metastasis.

In our previous work, this specific double mutation NICD GoF/p53 LoF has been carried out experimentally in a mouse model, by crossing the *villin-CreERT2* mice [129] (in this study referred as p53 LoF) and *RosaN1ic* mice [130] (in this study referred as NICD GoF) with the isogenic C57BL/6 animals to generate the NICD GoF/p53 LoF compound mice. These compound mice develop intestinal tumours with metastatic tumours to distal organs [31]. Our logical model successfully reproduces experimental observations of the compound mouse and proposes mechanisms explaining the metastatic phenotype with high penetrance in mice. In addition, we have investigated the role of TGF-β pathway in metastasis and showed its crucial role in the metastatic phenotype in the double mutant. Suppressing the TGF-β pathway might be an interesting target therapy to control metastasis, however future studies are required.

We also explored the activity of the Wnt pathway in the double mutant. Increased activity of the Wnt pathway due to mutations in the *apc* and *ctnnb1* genes leads to tumourigenesis of many cancers [131–133] and subsequently to metastasis [134,135]. Our mathematical model predicts phenotypes that correspond to adenocarcinomas as a result of linear progression of acquired mutations during sporadic colorectal cancer (CRC) suggested by the “Vogelstein sequence” [136] but no metastasis is reached with the model. Indeed, when we simulate APC LoF, KRAS GoF and p53 LoF (the Vogelstein sequence), the model predicts stable states of cells that are not arrested in the cell cycle, can undergo EMT and can invade (see S4 Table). Thus our logical model supports the hypothesis that the Wnt pathway contributes to tumour initiation [137]. However, there is still a debate if the Wnt pathway is actively involved in metastasis. For example, a negative correlation has been demonstrated between the presence of β-catenin and metastasis in breast cancer [138], in lung cancer [139–141], and in CRC [142–144]. It has been also demonstrated that the canonical Wnt pathway (β-catenin-dependent pathway) is suppressed at the leading edge of the tumour [145] and this might happen without affecting the β-catenin protein levels [146,147]. In the mouse model with Notch GoF /p53 LoF double mutation, in some tumours samples, mutations in *apc* and *ctnnb1* have been found but also tumours without those mutations have been shown to acquire metastasis. Both truncated APC and mutations in β-catenin correspond in our mathematical model to full activation of CTNNB1 and this will induce activation of AKT1. In our model, activation of AKT1 will inhibit migration and therefore inhibit metastasis. Appearance of metastasis in the mouse model with activated Wnt pathway might be putatively explained if one looks at the length of the truncated APC isoform for tumours with *apc* mutation. The APC mutation found in the Notch GoF /p53 LoF mouse model results in a relatively large truncated APC isoform that might still have inhibitory effect on β-catenin [148]. More details about the APC isoforms and its effect on β-catenin can be found in S3 Text.

Another explanation for having metastasis in tumours with active Wnt pathway might be the involvement of another mutation that affects the *akt1* or the *akt2* gene. According to our model, the Wnt pathway inhibits metastasis by up-regulation of AKT1. There are tumours in CRC patients (TCGA data from http://cbioportal.org, [31]) that can have an *akt2* gene amplification or a homozygous deletion or missense mutation of *akt1*. AKT2 induces migration while AKT1 inhibits migration thus the ratio AKT1 to AKT2 might be an important determinant for acquiring metastasis in the colon. Indeed studies have shown that AKT2 is predominant in sporadic colon cancer [149] and have a critical role in metastasis in CRC [150].

A Boolean model of EMT induction has been recently published, where the theoretical prediction that the Wnt pathway can be activated upon TGF-β administration was validated experimentally by measuring increased gene expression of the Wnt target gene *axin2* in Huh7 and PLC/PRF/5 cell lines [151]. Those cell lines are derived from hepatocellular carcinomas [152,153] and both can harbour known mutations [154] and unconfirmed mutations (http://tinyurl.com/l6mjd8y) that affect the signalling pathways: the Wnt pathway has constitutive activity in the Huh7 cell line [137,155]. An alternative explanation could be that our model is more specific for epithelial cancers as the model depicts many reactions observed in epithelial cells; it has been shown that different types of cancer have different protein (or isoforms) abundance [112,149]. Therefore, our model might be less adequate in predicting the activity for certain nodes for hepatocellular carcinoma and lung adenocarcinoma.

EMT is considered to be the first step and is very often modelled as an equivalent of having metastasis once it is activated. We provide here a logical model that proposes the involvement of three independent processes in order to have metastasis: EMT, invasion and migration. These phenotypes are controlled by an intricate network and only when EMT, invasion and migration do occur, can metastasis happen. The logical model explores the mechanisms and interplays between pathways that are involved in the processes, identifies the main players in these mechanisms and gives insight on how these pathways could be altered in a therapeutic perspective. Note that other mechanisms involving other alterations in the pathways that we model, or in other pathways might also take place, and we do not claim that our approach cover all possibilities of inducing metastasis. Still, our approach provides candidate intervention points for designing innovative anti-metastatic strategies.

## Supporting Information

S1 Text Review on published articles of mathematical models of EMT. (DOCX)

S2 Text Link between model solutions and transcriptomics data. (DOCX)

S3 Text Description of Wnt pathway. (PDF)

S4 Text From the master model to the reduced model. (DOCX)

S1 Fig Colon transcriptomics data. Mean value expression for each gene is mapped on the network. The figure is the same for both metastatic and non-metastatic samples.
(PNG)

S2 Fig Modular network of the metastasis model. (PDF)

S3 Fig Colon transcriptomics data mapped onto the modular network. The score for the modules are calculated based on the expression of target genes for metastatic and non-metastatic samples.
(PDF)

S4 Fig Mean gene expression value of the three replicates for the genes of the network at 4 different time points: At t = 0, at t = 8h, at t = 24h and at t = 72h. Green nodes correspond to low expression and red nodes to high expression. The minimum and maximum expression values are set over the whole dataset and are the same for the four graphs.
(PDF)

S5 Fig Distribution of pairs of genes of the mathematical model in the four-dimensional space of *Metastasis* probabilities, corresponding to four possible mutation type combinations LoF/LoF, LoF/GoF, GoF/LoF, GoF/GoF (here LoF is Loss-of-Function and GoF is Gain-of-Function). The image shows a two-dimensional projection onto a non-linear principal manifold from the space defined by four metastatic phenotype probabilities [*p*(LoF/LoF);*p*(GoF/GoF); *p*(LoF/GoF)+*p*(GoF/LoF);|*p*(LoF/GoF)-*p*(GoF/LoF)|]. Projection density is shown in the background by grey shading. The size of the node corresponds to the amplitude of the node pair (maximum difference in phenotype probability between the four mutants: LoF/LoF, GoF/GoF, GoF/LoF, LoF/GoF), such that the most sensitive (allowing control of phenotype to maximal degree) gene pairs correspond to bigger node sizes. Five clusters are identified: they correspond to five patterns which existence can be guessed from the symmetry considerations and which are shown on the right panels. 1a) Any GoF cancels the phenotype while double LoF can amplify it (14% of gene pairs); 1b) Any LoF cancels the phenotype while double GoF can amplify it (13%); 2a) Double GoF cancels the phenotype, double LoF or synthetic-dosage interaction can amplify it (23%); 2b) Double LoF cancels the phenotype, double GoF or synthetic-dosage interaction (LoF/GoF or GoF/LoF) can amplify it (16%); 3) Double LoF and double GoF cancel the phenotype, while synthetic-dosage interaction can amplify it (30%). TP53-NICD (top-left corner) mutant is an extreme example of group 3. NICD-AKT2 (bottom-left corner) is an extreme example of group 2b.
(PNG)

S6 Fig Results of robustness tests for the logical model with respect to small changes in the logical rules of the model. In the wild type logical model, for each logical rule, several "variant" models were created by changing one or two "OR" or "AND" operators to "AND" or "OR" operators respectively. The resulting distributions of phenotype probabilities over all such model modifications are shown.
(PDF)

S1 Table Annotations of the logical model. (DOCX)

S2 Table Phenotypes that can be reached by setting the activity of a single module or pathway to always ON (GoF: gain of function) or always OFF (LoF: loss of function). (XLSX)

S3 Table Phenotypes that can be reached by setting the activity of a single EMT regulator to always ON (GoF: gain of function) or always OFF (LoF: loss of function). EMT regulators: Snai1, Snai2, Zeb1, Zeb2, and Twist1.
(XLSX)

S4 Table Table of mutants. For each condition or mutation, all possible inputs are considered. Thus, all possible outputs corresponding to stable states are shown in this table (values for internal variables are not shown). The existence of a stable state in accordance with what has been published is enough to conclude that the mutant is validated: there exists a condition for which the model explains the experiments. The fact that other stable states exist shows that for some particular conditions, the stable state could be reachable. For instance, for NICD GoF, we see that a stable state with metastasis exits which has not been observed in experiments. However, for this stable state, all p53 family members are OFF, thus, it is a particular situation.
(DOCX)

S5 Table Signatures of module activity. (XLS)

S6 Table Robustness analysis; table of mutants for the logical stable states of the perturbed models. (XLSX)

S1 File Detailed and modular models in GINsim and MaBoSS formats. The zip file includes: Detailed model in GINsim format (SuppMat_Model_Master_Model.zginml), Modular model in GINsim format (SuppMat_Model_ModNet.zginml), SuppMat_MaBoSS_MasterModel.bnd (To simulate the model, MaBoSS needs to be downloaded from maboss.curie.fr and launched with the following command line:./MaBoSS -c SuppMat_MaBoSS_MasterModel.cfg -o SuppMat_MaBoSS_MasterModel SuppMat_MaBoSS_MasterModel.bnd), SuppMat_MaBoSS_MasterModel.cfg, SuppMat_MaBoSS_ModNet.bnd (To simulate the model, MaBoSS needs to be downloaded from maboss.curie.fr and launched with the following command line:./MaBoSS -c SuppMat_MaBoSS_ModNet.cfg -o SuppMat_MaBoSS_ModNet SuppMat_MaBoSS_ModNet.bnd), SuppMat_MaBoSS_ModNet.cfg.
(ZIP)

S2 File SuppMat_metastasis_mutants.cys. (ZIP)
