# Supplementary material for: Mathematical Modelling of Molecular Pathways Enabling Tumour Cell Invasion and Migration
Source: PLoS Comput Biol. 2015 Nov 3;11(11):e1004571. doi: 10.1371/journal.pcbi.1004571 (PMC4631357; doi:10.1371/journal.pcbi.1004571)
Supplement: S1 Text — (DOCX) [file pcbi.1004571.s001.docx]

# Published mathematical models

One group has developed a mathematical model to elucidate the effect of the ECM on cancer invasion [1]. Using a hybrid discrete and continuous model, the authors predicted that selective pressure conditioned by properties of tumour microenvironment affects invasion. Stringent conditions (low oxygen concentration) lead to invasion, while less stringent conditions lead to co-existence of aggressive with less aggressive phenotypes and tumours with smooth non-invasive fronts. These tumour characteristics are predicted to be reversible under the appropriate micro-environmental conditions. Also, the effect of the pH gradient on tumour invasion has been modelled with the contra-intuitive conclusion that less aggressive (having a higher pH in the micro-environment) tumours would invade faster. The authors conclude that a tumour depends on the micro-environment to invade, and that the fact that aggressive tumours decrease the pH would also decreases the number of supportive cells in the micro-environment [2]. Another more recent study modelled the interaction between tumour cells and macrophages by the secretion of CSF-1 and EGF, respectively. The tumour cells secrete CSF-1 to attract macrophages that in turn secrete EGF thereby stimulating tumour cells, leading to aggregation of both tumour cells and macrophages, and migrating together towards blood vessels. The model showed that the ratio between CSF-1 and EGF affects the number of tumour cells in aggregates [3]. Also, the involvement of Zeb and Snail proteins on EMT and their regulation by microRNAs (miRNA) has been modelled [4]. The miR34/Snai1 module acts as a noise-integrator and the miR200/ZEB module is a stable circuit acting as a ternary switch. A second continuous model addressed the possibility that Snai1 and Zeb1/2 are the core regulators of TGF-β-induced EMT. They showed that EMT is a sequential two-step program in which a cell goes from epithelial to partial EMT and subsequently to mesenchymal phenotype depending on the strength and duration of the TGF-β signal [5]. Both continuous models have been tested experimentally by measuring the dynamics of EMT and it has been shown that two-step EMT program predicted by both models occurred [6].

Both continuous models focus more on the reciprocal dynamics of the miRNAs and the EMT inducers Snai and Zeb protein families, while our model uses the Boolean formalism to model the possibility of having metastasis depending on the activity of EMT, invasion and migration. During the preparation of our manuscript, a study using logical modelling showing the effect of activated TGF-β pathway on EMT has been published.

A detailed Boolean model showed that a constitutive active TGF-β pathway activates EMT in hepatocellular carcinoma cells and EMT activation occurs together with activation of the Wnt and Hedgehog pathway [7]. Our Boolean model shows the effect of a mutation in the TGF-β pathway on EMT and metastasis but in addition, we show the effect of single and double mutations in other pathways on metastasis as a result of perturbed regulation of EMT, invasion and migration.

1. Anderson ARA, Weaver AM, Cummings PT, Quaranta V (2006) Tumor morphology and phenotypic evolution driven by selective pressure from the microenvironment. Cell 127: 905–915. doi:10.1016/j.cell.2006.09.042.

2. Martin NK, Gaffney EA, Gatenby RA, Maini PK (2010) Tumour-stromal interactions in acid-mediated invasion: a mathematical model. J Theor Biol 267: 461–470. doi:10.1016/j.jtbi.2010.08.028.

3. Knutsdottir H, Palsson E, Edelstein-Keshet L (2014) Mathematical model of macrophage-facilitated breast cancer cells invasion. J Theor Biol. doi:10.1016/j.jtbi.2014.04.031.

4. Lu M, Jolly MK, Levine H, Onuchic JN, Ben-Jacob E (2013) MicroRNA-based regulation of epithelial-hybrid-mesenchymal fate determination. Proc Natl Acad Sci U S A 110: 18144–18149. doi:10.1073/pnas.1318192110.

5. Tian X-J, Zhang H, Xing J (2013) Coupled reversible and irreversible bistable switches underlying TGFβ-induced epithelial to mesenchymal transition. Biophys J 105: 1079–1089. doi:10.1016/j.bpj.2013.07.011.

6. Zhang J, Tian X-J, Zhang H, Teng Y, Li R, et al. (2014) TGF-β-induced epithelial-to-mesenchymal transition proceeds through stepwise activation of multiple feedback loops. Sci Signal 7: ra91. doi:10.1126/scisignal.2005304.

7. Steinway SN, Gomez Tejeda Zañudo J, Ding W, Rountree CB, Feith DJ, et al. (2014) Network modeling of TGFβ signaling in hepatocellular carcinoma epithelial-to-mesenchymal transition reveals joint Sonic hedgehog and Wnt pathway activation. Cancer Res. doi:10.1158/0008-5472.CAN-14-0225.
