# Supplementary material for: Mathematical Modelling of Molecular Pathways Enabling Tumour Cell Invasion and Migration
Source: PLoS Comput Biol. 2015 Nov 3;11(11):e1004571. doi: 10.1371/journal.pcbi.1004571 (PMC4631357; doi:10.1371/journal.pcbi.1004571)
Supplement: S2 Text — (DOCX) [file pcbi.1004571.s002.docx]

# Link between model solutions and transcriptomics data

**Data downloaded in GEO Datasets:**

Accession: GDS3710

ID: 3710

PMID: 20007254

**Six steps:**

1. Identification of a list of genes (genes of the model)

(2) Average of the three replicates per time points

The values correspond to the average of the expression for the genes of the network. The values in yellow are genes with significant differential expression from time Tn vs. time T0 (p-value > 0.002 and FC > 2)

| Genes | Probes | T0 | T8 | T24 | T72 |
| --- | --- | --- | --- | --- | --- |
| AKT1 | 207163_s_at | 3.428702 | 3.507143 | 3.443265 | 3.401361 |
| AKT2 | 225471_s_at | 3.130943 | 3.160113 | 3.179635 | 3.196639 |
| CASP9 | 203984_s_at | 2.925062 | 2.759234 | 2.809112 | 2.763334 |
| CDC42 | 208727_s_at | 3.640598 | 3.565761 | 3.616913 | 3.343094 |
| CDH1 | 201130_s_at | 3.026043 | 2.817531 | 2.293341 | 2.218566 |
| CDH2 | 203440_at | 2.382185 | 2.93566 | 3.312995 | 3.508422 |
| CDKN1A | 202284_s_at | 3.604593 | 3.920846 | 3.954957 | 3.95623 |
| CTNNB1 | 1554411_at | 2.546302 | 2.907707 | 2.895337 | 3.297775 |
| DKK1 | 204602_at | 4.096997 | 4.295588 | 3.747287 | 3.606615 |
| EGFR | 210984_x_at | 2.429067 | 2.979868 | 3.497301 | 3.219565 |
| MAPK1 | 1552263_at | 2.524024 | 2.609834 | 2.610992 | 2.710342 |
| MMP2 | 201069_at | 2.353154 | 2.469234 | 3.230834 | 3.389101 |
| NOTCH1 | 218902_at | 2.911746 | 2.895011 | 2.803374 | 2.712926 |
| SMAD3 | 205397_x_at | 2.677022 | 2.942168 | 2.680722 | 2.294899 |
| SNAI1 | 219480_at | 2.369635 | 2.45224 | 2.618844 | 2.387523 |
| SNAI2 | 213139_at | 2.258083 | 2.91426 | 3.34554 | 3.590783 |
| TGFB1 | 203085_s_at | 2.427603 | 2.76206 | 2.872398 | 2.837521 |
| TP53 | 211300_s_at | 3.005923 | 3.06391 | 2.906158 | 2.828006 |
| TP73 | 1554379_a_at | 2.552107 | 2.523012 | 2.545394 | 2.413292 |
| TWIST1 | 213943_at | 2.101145 | 2.143635 | 2.177423 | 2.081249 |
| VIM | 1555938_x_at | 2.16561 | 2.627394 | 2.669708 | 2.792758 |
| ZEB1 | 210875_s_at | 2.569647 | 2.636942 | 2.808487 | 3.0069 |
| ZEB2 | 235593_at | 2.011638 | 2.109989 | 2.105275 | 2.197537 |

(3) Discretization of the data

Threshold of expression at 2.7

The threshold is thus set at 2.7. If the expression of the gene is above the threshold, it is equal to 1, if expression is below the threshold, it is equal to 0.

| Genes | T0_bool | T8_bool | T24_bool | T72_bool |
| --- | --- | --- | --- | --- |
| AKT1 | 1 | 1 | 1 | 1 |
| AKT2 | 1 | 1 | 1 | 1 |
| CASP9 | 1 | 1 | 1 | 1 |
| CDC42 | 1 | 1 | 1 | 1 |
| CDH1 | 1 | 1 | 0 | 0 |
| CDH2 | 0 | 1 | 1 | 1 |
| CDKN1A | 1 | 1 | 1 | 1 |
| CTNNB1 | 0 | 1 | 1 | 1 |
| DKK1 | 1 | 1 | 1 | 1 |
| EGFR | 0 | 1 | 1 | 1 |
| MAPK1 | 0 | 0 | 0 | 1 |
| MMP2 | 0 | 0 | 1 | 1 |
| NOTCH1 | 1 | 1 | 1 | 1 |
| SMAD3 | 0 | 1 | 0 | 0 |
| SNAI1 | 0 | 0 | 0 | 0 |
| SNAI2 | 0 | 1 | 1 | 1 |
| TGFB1 | 0 | 1 | 1 | 1 |
| TP53 | 1 | 1 | 1 | 1 |
| TP73 | 0 | 0 | 0 | 0 |
| TWIST1 | 0 | 0 | 0 | 0 |
| VIM | 0 | 0 | 0 | 1 |
| ZEB1 | 0 | 0 | 1 | 1 |
| ZEB2 | 0 | 0 | 0 | 0 |

(4) Identification of the stable states of the model

The number of stable states is not the same as the one presented in Table S4: the values of the internal nodes vary for different combinations of inputs. For example, the two metastatic stable states M1 and M2 seem to be the same in this table but, in fact, vary because one has DNA damage ON and the other one does not (not shown here).

| genes model | variables | HS | Apop1 | Apop2 | Apop4 | Apop5 | EMT1 | EMT2 | M1 | M2 |
| --- | --- | --- | --- | --- | --- | --- | --- | --- | --- | --- |
| AKT1 | AKT1 | 0 | 0 | 0 | 0 | 0 | 0 | 0 | 0 | 0 |
| AKT2 | AKT2 | 0 | 0 | 0 | 0 | 0 | 1 | 1 | 1 | 1 |
| CASP9 | Apoptosis | 0 | 1 | 1 | 1 | 1 | 0 | 0 | 0 | 0 |
| CDC42 | Migration | 0 | 0 | 0 | 0 | 0 | 0 | 0 | 1 | 1 |
| CDH1 | Cdh1 | 1 | 1 | 1 | 1 | 1 | 0 | 0 | 0 | 0 |
| CDH2 | Cdh2 | 0 | 0 | 0 | 0 | 0 | 1 | 1 | 1 | 1 |
| CDKN1A | p21 | 0 | 1 | 1 | 1 | 1 | 0 | 0 | 0 | 0 |
| CTNNB1 | CTNNB1 | 0 | 0 | 0 | 0 | 0 | 0 | 0 | 0 | 0 |
| DKK1 | DKK1 | 0 | 0 | 0 | 0 | 0 | 0 | 0 | 1 | 1 |
| EGFR | GF | 0 | 0 | 0 | 0 | 0 | 1 | 1 | 1 | 1 |
| MAPK1 | ERK | 0 | 0 | 0 | 0 | 0 | 1 | 1 | 1 | 1 |
| MMP2 | Invasion | 0 | 0 | 0 | 0 | 0 | 0 | 0 | 1 | 1 |
| NOTCH1 | NICD | 0 | 0 | 0 | 0 | 0 | 0 | 0 | 1 | 1 |
| SMAD3 | SMAD | 0 | 0 | 0 | 0 | 0 | 0 | 0 | 1 | 1 |
| SNAI1 | Snai1 | 0 | 0 | 0 | 0 | 0 | 1 | 1 | 1 | 1 |
| SNAI2 | Snai2 | 0 | 0 | 0 | 0 | 0 | 1 | 1 | 1 | 1 |
| TGFB1 | TGFbeta | 0 | 0 | 0 | 1 | 1 | 0 | 0 | 1 | 1 |
| TP53 | TP53 | 0 | 1 | 0 | 1 | 0 | 0 | 0 | 0 | 0 |
| TP73 | TP73 | 0 | 0 | 1 | 0 | 1 | 0 | 0 | 0 | 0 |
| TWIST1 | Twist1 | 0 | 0 | 0 | 0 | 0 | 1 | 1 | 1 | 1 |
| VIM | Vim | 0 | 0 | 0 | 0 | 0 | 1 | 1 | 1 | 1 |
| ZEB1 | Zeb1 | 0 | 0 | 0 | 0 | 0 | 1 | 1 | 1 | 1 |
| ZEB2 | Zeb2 | 0 | 0 | 0 | 0 | 0 | 1 | 1 | 1 | 1 |

(5) Computation of a similarity matrix per time point and per stable state

We compare the discretized data to the table of stable states, and for each stable state and for each gene: For a given stable state, if a gene has the same value in the table of data at a particular time point and in the column corresponding to that stable state, the value is set to 1 in the similarity matrix. For example, for T0_bool (discretized vector of expression data at time T0), AKT1 is equal to 1 and AKT2 is equal to 1. The vector corresponding to the stable state “EMT1” shows AKT1 is equal to 0 and AKT2 is equal to 1. In the similarity matrix, the entry for AKT1 will be set to 0 (AKT1 value is different in EMT1 and in T0_bool) and the entry for AKT2 will be set to 1 (AKT2 has the same value in EMT1 and in T0_bool.

(6) Computation of scores for each stable state from the similarity matrix

A score for each of the stable state for each time point is computed. It corresponds to the sum of the similarities between a stable state and the data at a time point. In yellow, are highlighted the 3 highest scores per time point. The higher the value, the closer the steady state to the sample is.
