# Supplementary material for: Mathematical Modelling of Molecular Pathways Enabling Tumour Cell Invasion and Migration
Source: PLoS Comput Biol. 2015 Nov 3;11(11):e1004571. doi: 10.1371/journal.pcbi.1004571 (PMC4631357; doi:10.1371/journal.pcbi.1004571)
Supplement: S3 Text — (PDF) [file pcbi.1004571.s003.pdf]

Discussion on Wnt Pathway activity in metastasis

APC truncated mutations

The size of the truncated protein has an influence on the numbers of polyps. For example the APC^{min} mutant in which the *apc* gene harbours an additional stop codon at codon 850 results in high amounts of polyps (~120) in the intestine [1] while a truncation at codon 716 (APC^{716}) can give rise even to more than 300 polyps in the small intestine [2]. On the contrary, a longer truncated APC isoform, truncated at stop codon 1638, of APC^{1638N} may only give rise to 3 polyps [3] indicating that a larger truncated form of APC still harbours an inhibitory activity due to the β-catenin inhibitory domain (CID) which is located in the mutation cluster region (MCR) and can thus be found in many truncated APC's found in human tumours [4,5]. These truncated forms still contain sufficient amino acid repeats to phosphorylate β-catenin followed by degradation by the destruction complex [5,6]. Furthermore, it has been described that tumours with APC LoF mutations do require canonical wnt ligands in order to have activated Wnt pathway activity [7,8]. Compared to wild type APC, the phosphorylation of β-catenin capacity seems to be relatively weak but highly active, thus still having significant impact on the regulation of β-catenin in cancer cells [6]. It has been demonstrated that silencing of truncated APC leads to increased β-catenin-mediated transcriptional activity and β-catenin protein level [9] which is in accordance that truncated APC can phosphorylate β-catenin for degradation.

As for mutation of β-catenin, it occurs even less frequently than mutations of both APC alleles [10] and it has been shown that in Wilms tumours, mutational-activated β-catenin induces transcription of mainly genes involved in proliferation or genes that code for proteins involved in the inhibition of the Wnt pathway [11]. To conclude, in our modelling framework, we can only account for full inhibition or activation of genes. More refined modelling might be needed to account for these forms of truncated APC and mutations.

1. Moser AR, Pitot HC, Dove WF (1990) A dominant mutation that predisposes to multiple intestinal neoplasia in the mouse. Science 247: 322–324.

2. Fodde R, Edelmann W, Yang K, van Leeuwen C, Carlson C, et al. (1994) A targeted chain-termination mutation in the mouse Apc gene results in multiple intestinal tumors. Proc Natl Acad Sci U S A 91: 8969–8973.
3. Taketo MM (2006) Mouse models of gastrointestinal tumors. Cancer Sci 97: 355–361. doi:10.1111/j.1349-7006.2006.00190.x.
4. Kohler EM, Chandra SHV, Behrens J, Schneikert J (2009) Beta-catenin degradation mediated by the CID domain of APC provides a model for the selection of APC mutations in colorectal, desmoid and duodenal tumours. Hum Mol Genet 18: 213–226. doi:10.1093/hmg/ddn338.
5. Schneikert J, Grohmann A, Behrens J (2007) Truncated APC regulates the transcriptional activity of beta-catenin in a cell cycle dependent manner. Hum Mol Genet 16: 199–209. doi:10.1093/hmg/ddl464.
6. Wang L, Liu X, Gusev E, Wang C, Fagotto F (2014) Regulation of the phosphorylation and nuclear import and export of β-catenin by APC and its cancer-related truncated form. J Cell Sci 127: 1647–1659. doi:10.1242/jcs.131045.
7. Scholer-Dahirel A, Schlabach MR, Loo A, Bagdasarian L, Meyer R, et al. (2011) Maintenance of adenomatous polyposis coli (APC)-mutant colorectal cancer is dependent on Wnt/beta-catenin signaling. Proc Natl Acad Sci U S A 108: 17135–17140. doi:10.1073/pnas.1104182108.
8. Voloshanenko O, Erdmann G, Dubash TD, Augustin I, Metzig M, et al. (2013) Wnt secretion is required to maintain high levels of Wnt activity in colon cancer cells. Nat Commun 4: 2610. doi:10.1038/ncomms3610.
9. Chandra SHV, Wacker I, Appelt UK, Behrens J, Schneikert J (2012) A common role for various human truncated adenomatous polyposis coli isoforms in the control of beta-catenin activity and cell proliferation. PLoS One 7: e34479. doi:10.1371/journal.pone.0034479.
10. Rowan AJ, Lamlum H, Ilyas M, Wheeler J, Straub J, et al. (2000) APC mutations in sporadic colorectal tumors: A mutational "hotspot" and interdependence of the "two hits". Proc Natl Acad Sci U S A 97: 3352–3357.
11. Zirn B, Samans B, Wittmann S, Pietsch T, Leuschner I, et al. (2006) Target genes of the WNT/beta-catenin pathway in Wilms tumors. Genes Chromosomes Cancer 45: 565–574. doi:10.1002/gcc.20319.
