# Supplementary material for: Mathematical Modelling of Molecular Pathways Enabling Tumour Cell Invasion and Migration
Source: PLoS Comput Biol. 2015 Nov 3;11(11):e1004571. doi: 10.1371/journal.pcbi.1004571 (PMC4631357; doi:10.1371/journal.pcbi.1004571)
Supplement: S4 Text — (DOCX) [file pcbi.1004571.s004.docx]

# From Master Model (Master) to Modular Model (ModNet)

1. In GINsim, export the network of the Master Model to Cytoscape format (xgmml)
2. Install experimental version of BiNoM Plugin in Cytoscape (version 2.8.x) from website: binom.curie.fr
3. Import Master Model in Cytoscape
4. Create subnetworks of each module (even single nodes) by duplicating the master network and deleting the extra nodes for each module:
   1. ECMicroenv
   2. DNAdamage
   3. GF
   4. Notch_pthw (NICD)
   5. TGFb_pthw (TGFbeta, SMADs)
   6. Wnt_pthw (DKK1, CTNNB1)
   7. ERK_pthw (ERK)
   8. E-cadh (Cdh1)
   9. EMTreg (TWIST1, ZEB1, ZEB2, SNAI1, SNAI2, CDH2)
   10. AKT1
   11. AKT2
   12. p53
   13. p63_p73 (p63, p73)
   14. miRNA (miR203, miR200, miR34)
   15. EMT
   16. Invasion
   17. Migration
   18. Metastasis
   19. CCA (CellCycleArrest, p21)
   20. Apoptosis
5. Create a network of the defined modules
   1. Plugins => BiNoM 2.5 => Module Manager => Create Networks of Modules
   2. Plugins => BiNoM 2.5 => Module Manager => Create Links between Modules
6. Verify the coherence of the network by listing the links proposed by BiNoM between modules
   1. Plugins => BiNoM 2.5 => Module Manager List Edges between Modules
   2. Copy and paste in Excel (for instance)
7. Make choices

For each edge in the Master Model, the corresponding edge for the modular network is provided. If all edges going from one module to another are positive (or negative), the edge between the modules is positive (or negative). In some cases, some choices have to be made:

- Ambiguous cases

Most of the edges are coherent except for two cases where there are ambiguous choices:

p63_p73 => miRNA

p63_p73 =| miRNA

corresponding to the two edges:

p63 => miR200

p63 =| miR34

and:

AKT2 => miRNA

AKT2 =| miRNA

corresponding to:

AKT2 => miR200

AKT2 =| miR34

The choice between activating or inhibiting edges was made based on the similarities with the solutions of the master model. Thus, the choice to keep the following rules was made:

p63_p73 => miRNA

AKT2 => miRNA

Ignoring thus the influences on miR34 at the level of the module.

- Deletion of edges.

To match the solutions of the master model, other choices needed to be made

- - miRNA =| p63_p73 (miR203 =| p63)
  - miRNA => p53 (miR203 => p53)

This leads to the choice of miR200 being the most important component of the module miRNA.

1. List the logical rule

The logical rules corresponding to the reduced model are the following (with the variables: ECMicroenv and DNAdamage as inputs):

| Node | Rule |
| --- | --- |
| AKT1 | WNT_pthw & (Notch_pthw \| TGFb_pthw \| GF \| EMTreg) & !miRNA & !p53 & !Ecadh |
| AKT2 | (TGFb_pthw \| GF \| Notch_pthw \| EMTreg) & EMTreg & !miRNA & !p53 |
| Ecadh | !AKT2 & !EMTreg |
| WNT_pthw | !Notch_pthw & !EMTreg & !miRNA & !p53 & !p63_73 & !AKT1 & !Ecadh & !WNT_pthw |
| ERK_pthw | (TGFb_pthw \| Notch_pthw \| GF \| EMTreg) & !AKT1 |
| GF | (GF \| EMTreg) & !Ecadh |
| miRNA | (p53 \| p63_73) & !AKT2 & !EMTreg & !AKT1 |
| Notch_pthw | ECMicroenv & !p53 & !p63_73 & !miRNA |
| p53 | (Notch_pthw \| DNAdamage \| WNT_pthw) & !AKT1 & !AKT2 & !p63_73 & !EMTreg |
| p63-73 | !Notch_pthw & !p53 & DNAdamage & !AKT2 & !AKT1 & !EMTreg |
| TGFb_pthw | (Notch_pthw \| ECMicroenv) & !WNT_pthw & !miRNA |
| EMTreg | (Notch_pthw \| WNT_pthw \| EMTreg) & !miRNA & !p53 |
| CCA | (((p53 \| p63_73 \| (TGFb_pthw & Notch_pthw) \| AKT2) & !ERK_pthw) \| miRNA \| EMTreg) & !AKT1 |
| Apoptosis | !ERK_pthw & !AKT1 & !EMTreg & (miRNA \| p63_73 \| p53) |
| EMT | !Ecadh & EMTreg |
| Invasion | (TGFb_pthw & EMTreg) \| WNT_pthw |
| Migration | EMT & ERK_pthw & AKT2 & Invasion & !AKT1 & !miRNA & !p63_73 |
| Metastasis | Migration |

1. Compare the stable states of the master model and the reduced model

- GINsim compares the stable state solutions of the two models


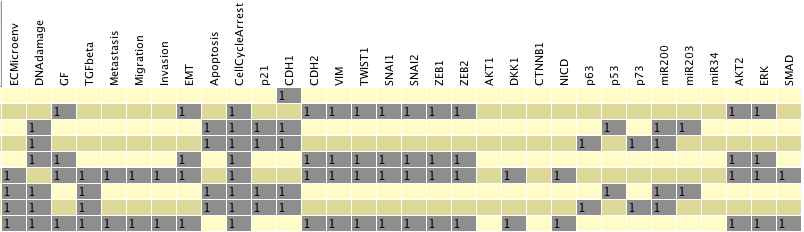


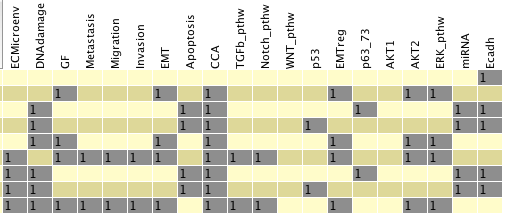


- Functionality of circuits

The reduction is particularly useful when studying the functionality of the circuits of the model or when the construction of the transition graph is needed to identify the existence of cyclic attractors. In this model, there are eight positive functional circuits and two negative functional circuits. Knowing that negative circuits can give rise to limit cycle attractors that are not reflected in stable state solution space, we simulated the conditions for the functionality of the negative circuits using GINsim features and observed that none of the two negative circuits (one involving p53 and Notch_pthw and another one involving an auto-regulation on WNT_pthw) leads to cyclic attractors. We confirmed below the results with MaBoSS.

- MaBoSS

We verified the probabilities to reach phenotypes for all random initial conditions using MaBoSS software.

Master Model

Modular Model

The two models are very similar. Because of the reduction, we expect some small differences in mutant phenotypes, but overall, we can conclude that the two models exhibit the same behaviours.

1. Pathway alterations versus node alterations

Note that when performing a pathway alteration in the reduced model corresponds to altering one (or more than one) gene(s) composing the module. Most of the modules include only one single node, for them, we can claim both alterations in the reduced and the master models are exactly the same. However, a pathway alteration may not be equivalent to a node alteration, e.g., a CTNNB1 overexpression or a Wnt pathway overexpression might not be the same since Wnt pathway module contains two nodes with opposite roles. We looked more particularly at the effect of some mutations in the EMT regulators module because it is composed of many nodes (S3_Table_EMT_mutants).

For an alteration in the EMTreg module, either EMT regulator can be altered. However, the analysis of the EMT module independently shows that Twist1, Snai1 and Zeb2 alterations have a greater effect on the outcome than the other EMT regulators.

For all these reasons, we do not claim that the two models, the master and the reduced models, are equivalent in the analysis of alterations, but they behave similarly in the wild type case and in mutants for which the modules contain one single node.

Note that our approach is a little different than most reduction techniques as it is the case for Naldi et al. (Theor. Comp. Sci. 412 (21), 2207-2218, 2011), for example, which reduces the number of variables propagating the changes into the functions rather than what we do which corresponds to creating modules and deducing rules based on the rules of the nodes that compose the module as explained in this document.
