# Supplementary figures and images for: Mathematical Modelling of Molecular Pathways Enabling Tumour Cell Invasion and Migration
Source: PLoS Comput Biol. 2015 Nov 3;11(11):e1004571. doi: 10.1371/journal.pcbi.1004571 (PMC4631357; doi:10.1371/journal.pcbi.1004571)

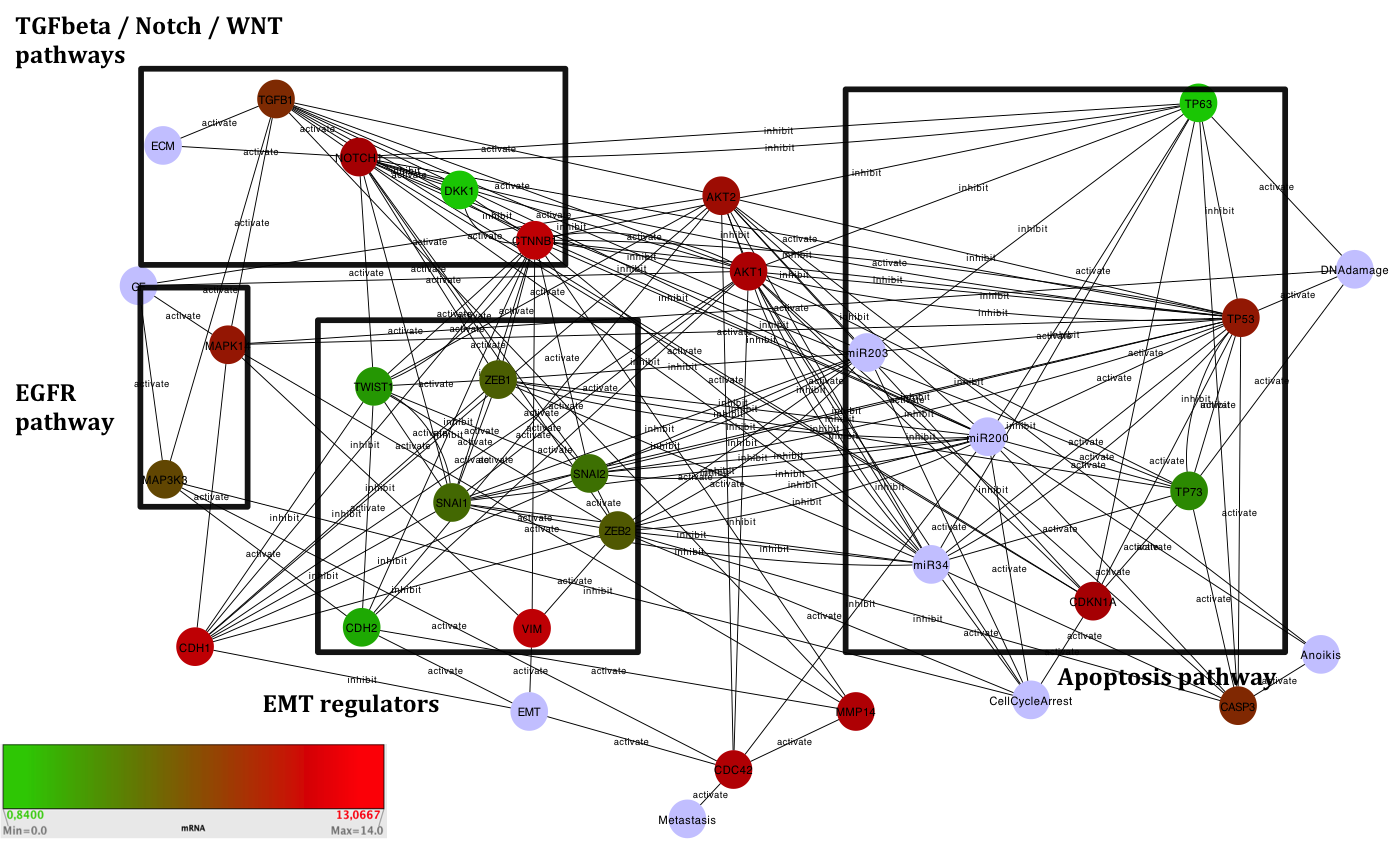

Supplement: S1 Fig — Mean value expression for each gene is mapped on the network. The figure is the same for both metastatic and non-metastatic samples. (PNG) [file pcbi.1004571.s005.png]

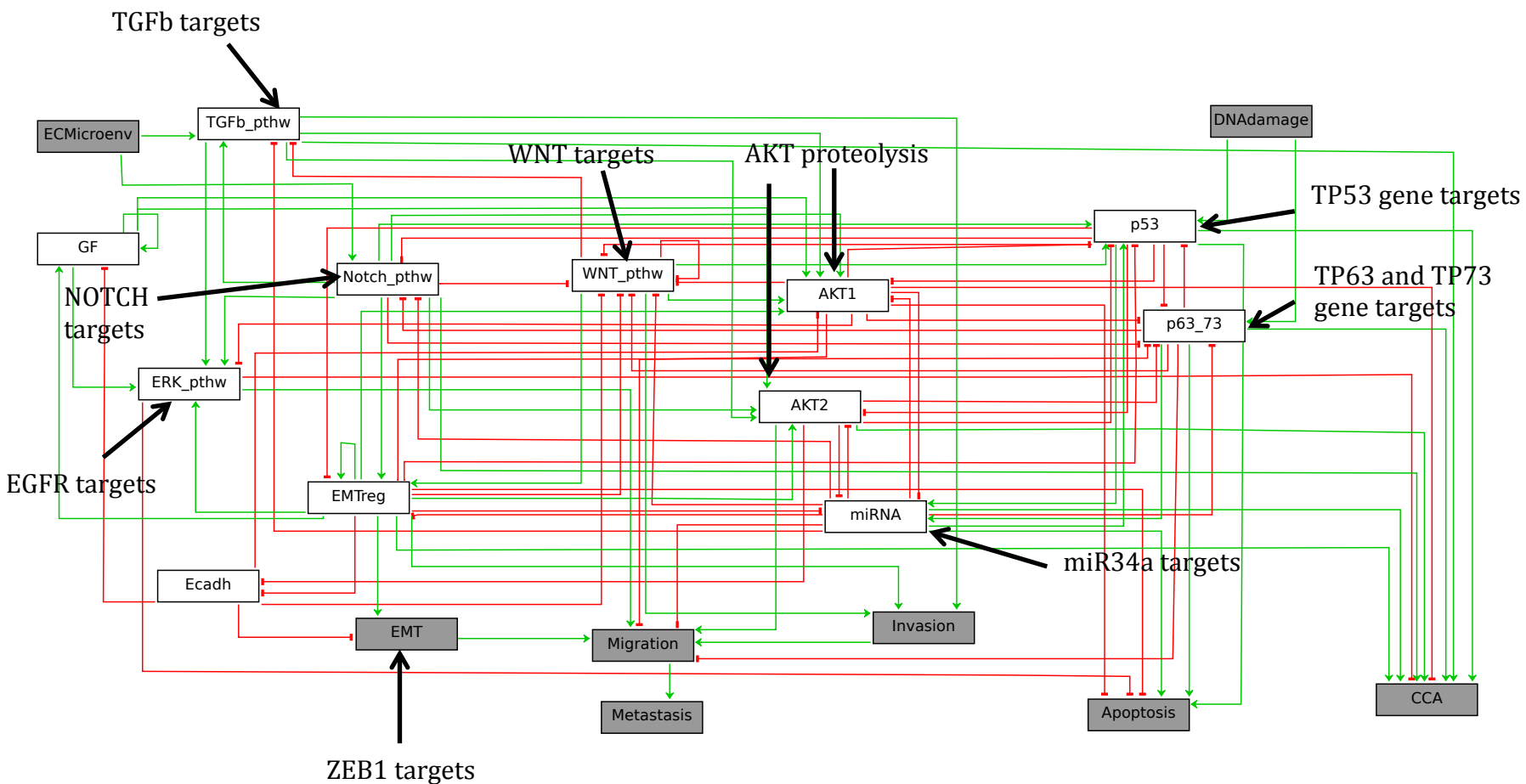

Supplement: S2 Fig — (PDF) [file pcbi.1004571.s006.pdf]

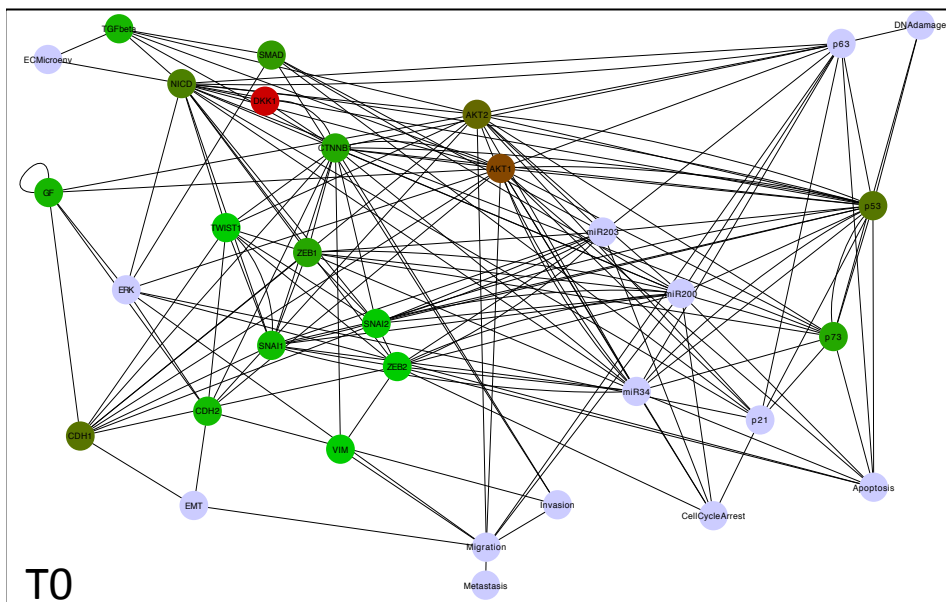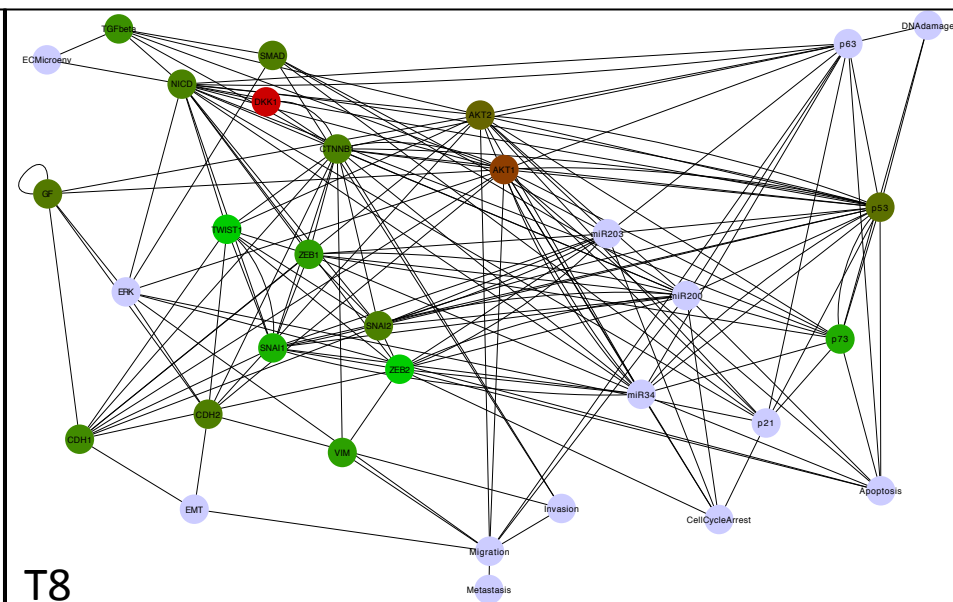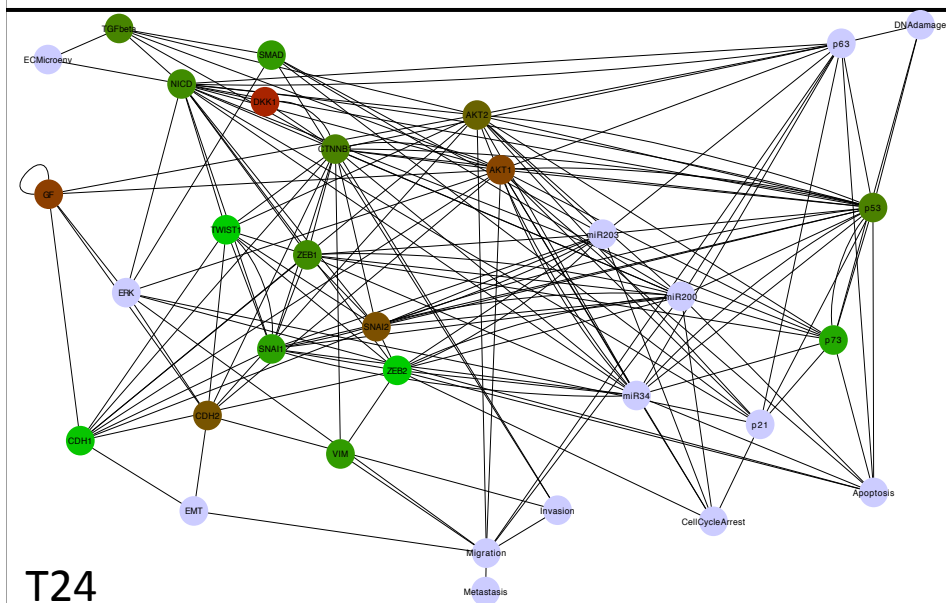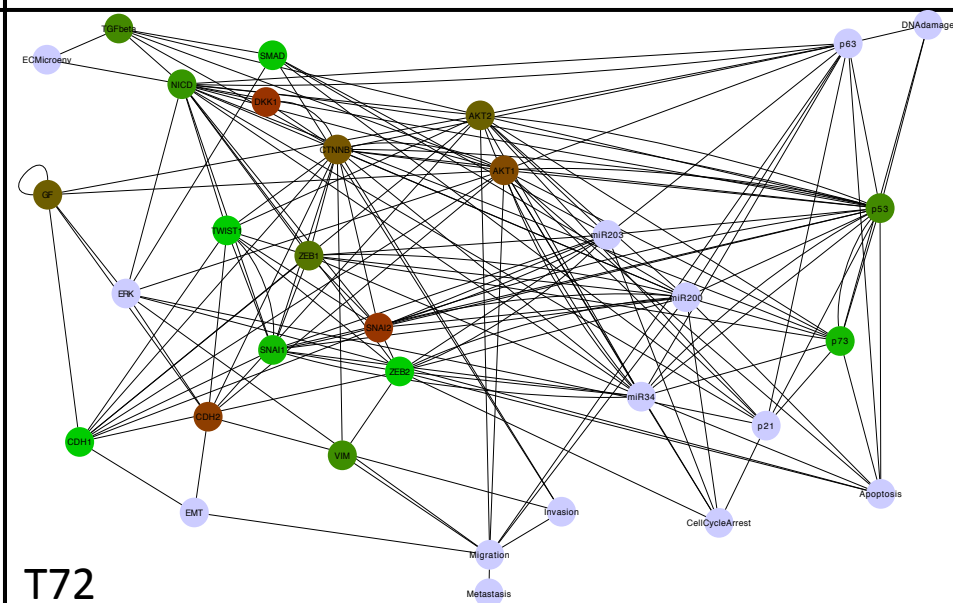

Supplement: S4 Fig — Green nodes correspond to low expression and red nodes to high expression. The minimum and maximum expression values are set over the whole dataset and are the same for the four graphs. (PDF) [file pcbi.1004571.s008.pdf]

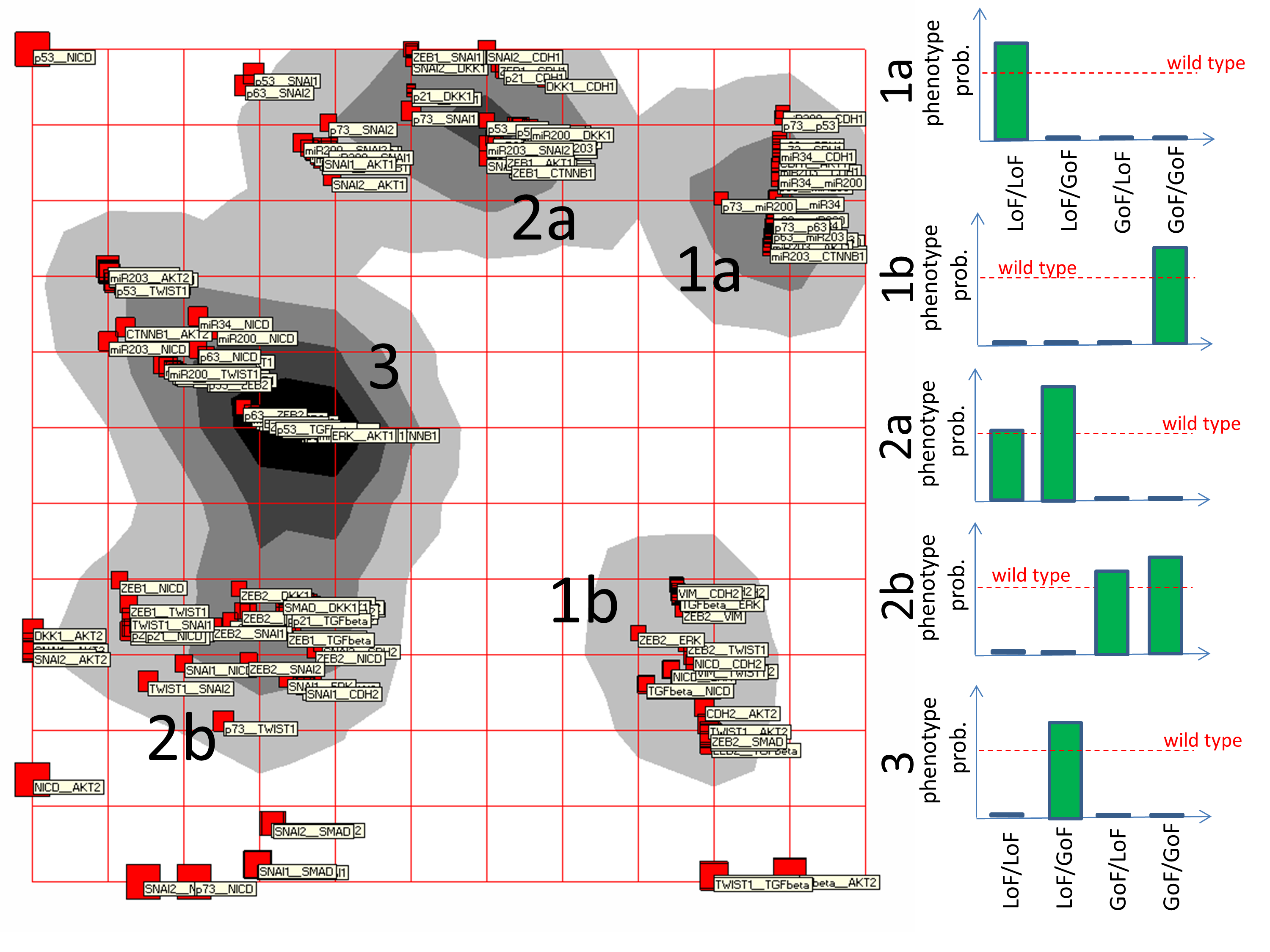

Supplement: S5 Fig — The image shows a two-dimensional projection onto a non-linear principal manifold from the space defined by four metastatic phenotype probabilities [p(LoF/LoF);p(GoF/GoF); p(LoF/GoF)+p(GoF/LoF);|p(LoF/GoF)-p(GoF/LoF)|]. Projection density is shown in the background by grey shading. The size of the node corresponds to the amplitude of the node pair (maximum difference in phenotype probability between the four mutants: LoF/LoF, GoF/GoF, GoF/LoF, LoF/GoF), such that the most sensitive (allowing control of phenotype to maximal degree) gene pairs correspond to bigger node sizes. Five clusters are identified: they correspond to five patterns which existence can be guessed from the symmetry considerations and which are shown on the right panels. 1a) Any GoF cancels the phenotype while double LoF can amplify it (14% of gene pairs); 1b) Any LoF cancels the phenotype while double GoF can amplify it (13%); 2a) Double GoF cancels the phenotype, double LoF or synthetic-dosage interaction can amplify it (23%); 2b) Double LoF cancels the phenotype, double GoF or synthetic-dosage interaction (LoF/GoF or GoF/LoF) can amplify it (16%); 3) Double LoF and double GoF cancel the phenotype, while synthetic-dosage interaction can amplify it (30%). TP53-NICD (top-left corner) mutant is an extreme example of group 3. NICD-AKT2 (bottom-left corner) is an extreme example of group 2b. (PNG) [file pcbi.1004571.s009.png]
