# Supplementary material for: Mathematical Modelling of Molecular Pathways Enabling Tumour Cell Invasion and Migration
Source: PLoS Comput Biol. 2015 Nov 3;11(11):e1004571. doi: 10.1371/journal.pcbi.1004571 (PMC4631357; doi:10.1371/journal.pcbi.1004571)
Supplement: S3 Fig — The score for the modules are calculated based on the expression of target genes for metastatic and non-metastatic samples. (PDF) [file pcbi.1004571.s007.pdf]

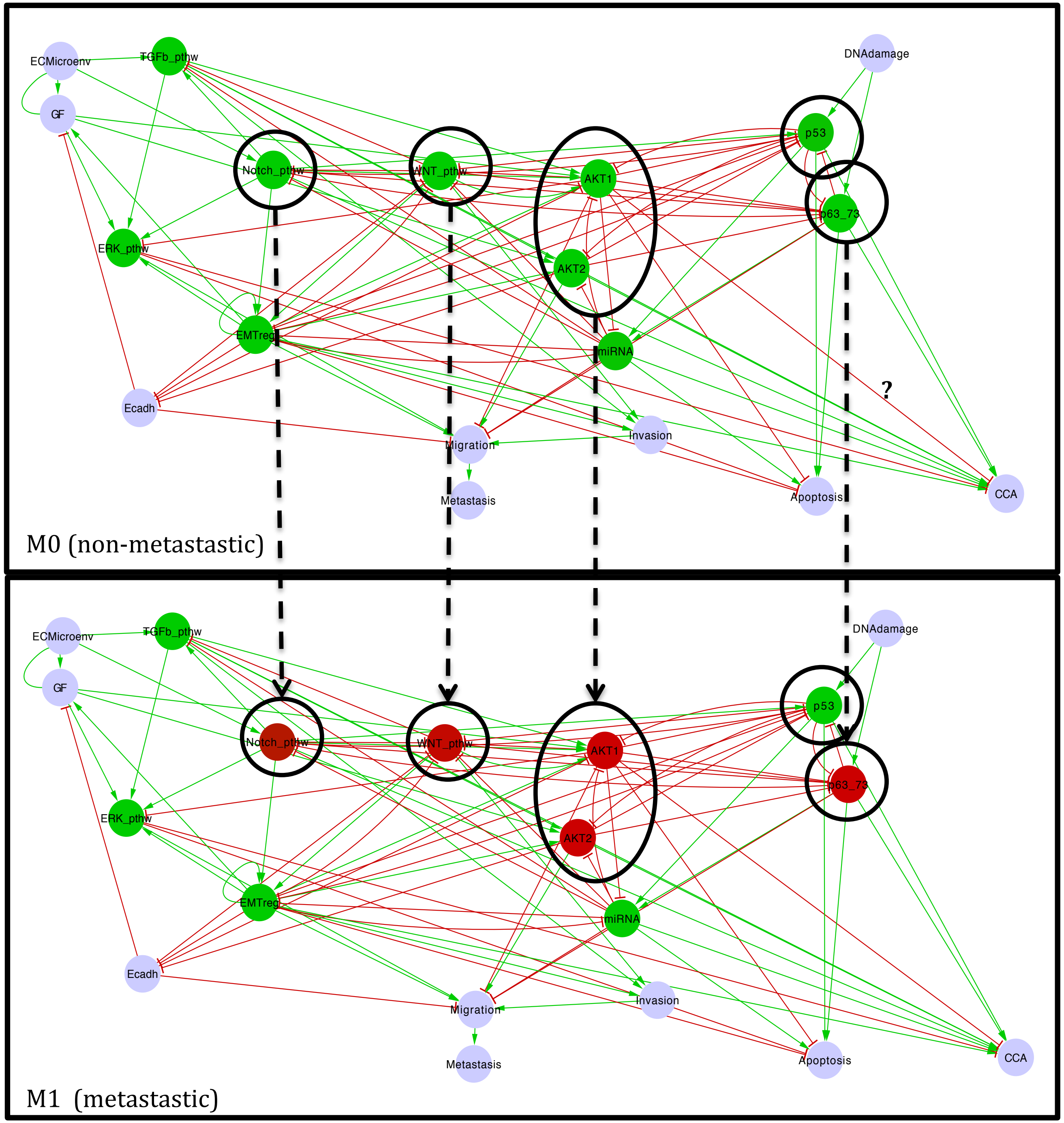

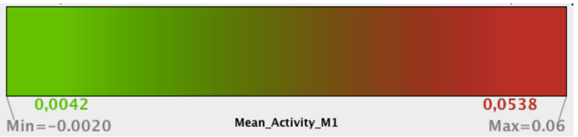

ModuleActivity	Mean_Activity_M0	Mean_Activity_M1
AKT1	-0.0036	0.0621
AKT2	-0.0036	0.0621
ERK_pthw	-8.00E-04	-0.0427
Notch_pthw	-0.0113	0.0479
TGFb_pthw	-0.0022	-0.0397
WNT_pthw	-0.0088	0.0516
p53	0.0066	-0.0577
p63_73	-0.0096	0.0767
miRNA	0.0059	-0.0496
EMTreg	-0.0056	-4.00E-04

Colon transcriptomics data mapped onto the modular network. The score for the modules are calculated based on the expression of target genes for metastastic and non-metastastic samples

We used the same data as the one described above for colon cancer samples. We tested target gene sets selected from MSigDB and KEGG databases together with several gene sets assembled by us from external sources (publications, ...). Among several available gene sets, we chose the ones having the best differential activity scoring between metastatic and non-metastatic samples.
