# Supplementary material for: Mathematical Modelling of Molecular Pathways Enabling Tumour Cell Invasion and Migration
Source: PLoS Comput Biol. 2015 Nov 3;11(11):e1004571. doi: 10.1371/journal.pcbi.1004571 (PMC4631357; doi:10.1371/journal.pcbi.1004571)
Supplement: S6 Fig — In the wild type logical model, for each logical rule, several "variant" models were created by changing one or two "OR" or "AND" operators to "AND" or "OR" operators respectively. The resulting distributions of phenotype probabilities over all such model modifications are shown. (PDF) [file pcbi.1004571.s010.pdf]

Analysis of all model variants, different from WT by at most 2 changes in logical operators (in one or two rules)

Metastasis phenotype probability

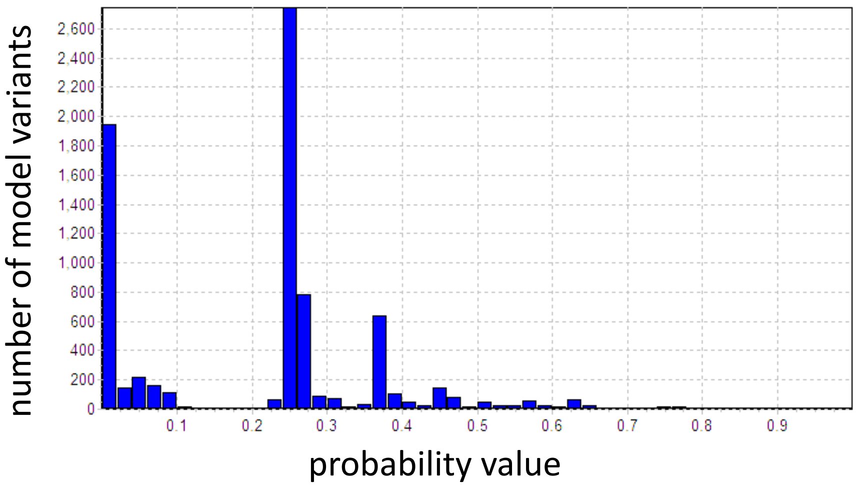

wild type probability for Metastasis = 0.26

EMT phenotype probability

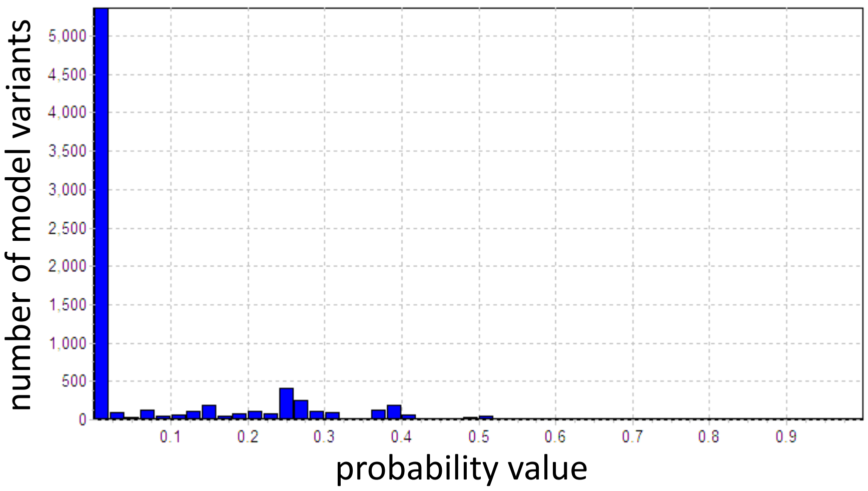

wild type probability for EMT = 0.01

Apoptosis phenotype probability

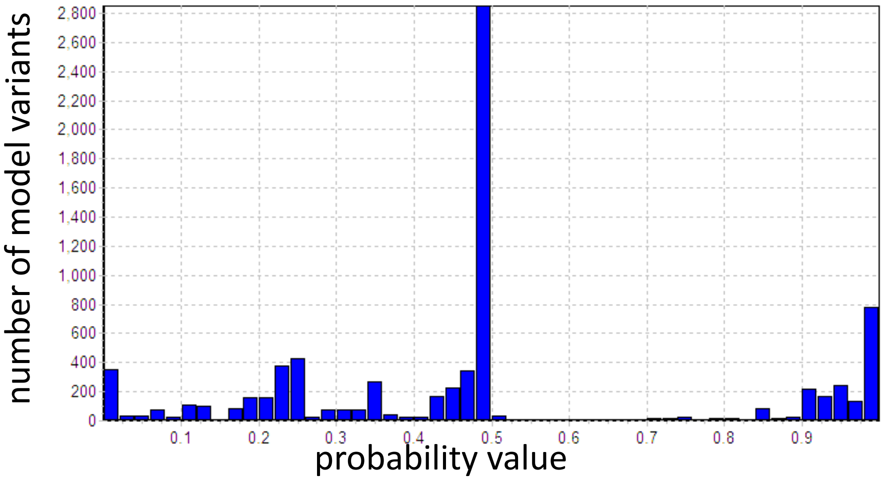

wild type probability for Apoptosis = 0.49

Cell Cycle Arrest phenotype probability

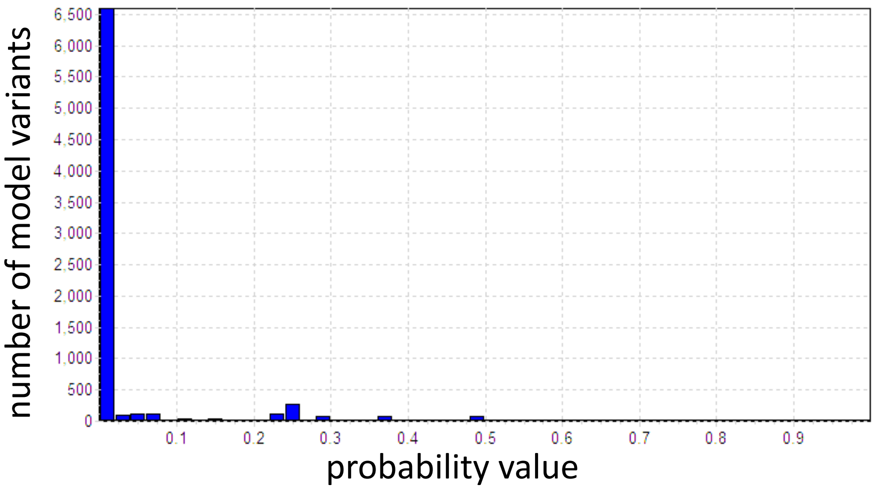

wild type probability for Cell Cycle Arrest = 0
