# Supplementary material for: Mathematical Modelling of Molecular Pathways Enabling Tumour Cell Invasion and Migration
Source: PLoS Comput Biol. 2015 Nov 3;11(11):e1004571. doi: 10.1371/journal.pcbi.1004571 (PMC4631357; doi:10.1371/journal.pcbi.1004571)
Supplement: S1 Table — (DOCX) [file pcbi.1004571.s011.docx]

| **Target node** | **Interaction description** | **Cell type** |
| --- | --- | --- |
| Migration | AKT2 induces beta integrins -> migration | Breast and ovarian cancer cells [1] |
|  | Inhibition of AKT2 compromises migration | Mesenchymal stem cell [2] |
|  | AKT1 inhibits migration through NFAT inhibition | Breast cancer cell line [3,4] |
|  | AKT1 inhibits migration through paladin | Breast cancer cell line [5] |
|  | ERK stimulates migration through cofilin | Rat fibroblast [6] |
|  | miR200 inhibits migration through targeting fibronectin1 | Breast cancer and endometrial cell line [7] |
|  | ERK activation is necessary for migration induced by AKT1 down- regulation | Breast epithelial cells [8] |
| Invasion | TGF-b induces MMP-2 and MMP-9 expresion | Breast epithelial and mammary adenocarcinoma cell line [9,10] |
|  | CTNNB1 induces transcription of MMPs | Keratinocytes, endothelial cells, murine mammary epithelial cell, T cell, chondrocytes nd human embryonic kidney cells [11–16] |
|  | N-cadherin is required for TGF-β induced invasion | Myofibroblast [17] |
| EMT | EMT: absence of epithelial markers and presence of mesenchymal markers | Avian epiboly, canine kidney cells, amniote embryos [18–20] |
| Apoptosis | p53 -> anoikis | Breast epithelial cells [21] |
|  | AKT1 -\| anoikis | Intestinal epithelial cells [22] |
|  | p63 -\| anoikis via integrin beta4 | (primary) human mammary epithelial cells [23] |
|  | miR34 -> apoptosis (by inhibiting anti-apoptotic gene BCL2) | Intestinal epithelium cells [24] |
|  | miR200 -\| XIAP | Gastric adenocarcinoma [25] |
|  | ERK inhibits apoptosis through Bcl2, Bcl-XL and Mcl-1 | Pancreatic adenocarcinoma [26] |
| CellCycleArrest | miR34 inhibits cell cycle progression by inhibiting Cyclin D1 activity. | Human breast adenocarcinoma [27] |
|  | miR203 inhibits cell cycle by targeting CDK6 | hepatocellular carcinoma [28] |
|  | miR200 -\| CCNE2 | Lymphoma, HEK cell line [29] |
|  | Only AKT1 is required for proliferation | Mouse myoblast and human fibroblast[30] |
|  | Zeb2 -\| CyclinD | Bladder cancer [31] |
|  | ERK activates proliferation | Myofibroblast [6] |
|  | miR34 -> cell cycle (E2F genes) | Colorectal cancer [24] |
| p21 | p63 -> p21 | Lung carcinoma [32–34] |
|  | p53 -> CellCycleArrest | Fibroblast [35,36] |
|  | p73 -> CellCycleArrest | Lung carcinoma [33,34] |
|  | Notch is required for growth arrest by TGF-β | mammary epithelial cells [37] |
|  | Snai1 and Twist cooperate in inhibition of p21 expression | osteosarcoma osteoblast-like cell, fibroblast [38] |
|  | AKT2 induces cell cycle arrest by keeping p21 into the nucleus | Mouse myoblast and human fibroblast, myoblast [30,39] |
| CDH1 | AKT1 down-regulation repressed CDH1 expression | Breast epithelial cells [8] |
|  | Snai1 inhibits CDH1 but requires AKT2 for it | Intestinal epithelial cells[40] |
|  | AKT2 -> Dab2 -\| CDH1 | Breast adenocarcinoma [41] |
|  | AKT2 require Snai1 to inhibit CDH1 | Intestinal epithelial cells [40] |
|  | Synergistic effect of Zeb1 & Snai2 on repression CDH1 | Keratinocytes [42] |
|  | Zeb1 and Zeb2 are required to collaborate with Snai2 to repress CDH1 | Mammary epithelial cells [43] |
|  | Twist1 and Snai1 cooperation to repress CDH1 | Mammary epithelial cells [43] |
| CDH2 | Twist activates transcription of CDH2 | Observed in many types of cancers [44] |
|  | shSnai2 has no effect on CDH2 gene expression | Keratinocytes [45] |
| VIM | CTNNB1->VIM | Breast cancer [46] |
|  | ZEB2-> VIM transcriptionally | Mammary epithelial and intestinal cells [47,48] |
|  | Snai2 not involved for VIM expression | Mammary epithelial cells [43] |
|  | Twist1 not involved for VIM expression | Mammary epithelial cells [49] |
| TWIST1 | NICD->Twist1 transcriptionally | Gastric adenocarcinoma and human embryonic kidney cells [50] |
|  | CTNNB1->Twist1 transcriptionally | Colon cancer [51] |
|  | Snai1->Twist1 | Mouse mammary epithelial cells [52] |
| SNAI1 | NICD->Snai1 | Human ovarian carcinoma [53] |
|  | Twist1->Snai1 | Mammary epithelial cells [43] |
|  | AKT->Snai1 | Keratinocytes [45] |
|  | miR203-\|Snai1 | Human breast cancer cell line [54] |
|  | miR34-\| Snai1 | Colon adenocarcinoma [55] |
|  | p53-\|Snai1 via complex formation with mdm2 | Hepatocellular carcinoma [56] |
|  | CTNNB1 -> miR-30e -\| Snai1 | Rat intestinal epithelial cells, non-small cell lung cancer [57,58] |
| SNAI2 | Twist1->Snai2 | Mammary epithelial cells [43] |
|  | b-catenin->Wnt pathway->Snai2 | Embryonic ectoderm [59] |
|  | NICD->Snai2 | Human breast epithelial cells, Human endothelial cell line [60,61] |
|  | miR200-\|Snai2 | AC3 cell line [62] |
|  | p53-\|Snai2 via mdm2 | Breast cancer cell line, non-small-cell lung cancer [63] |
|  | miR203 -\| Snai2 | Prostate cell lines EP156T and EPT1 [64] |
| ZEB1 | Snai1 and Twist1 cooperate to activate transcription of Zeb1 | Mouse mammary epithelial cells [52] |
|  | Snai2 -> Zeb1 | Keratinocytes [42] |
|  | CTNNB1->Zeb1 | Tumour c olorectal tissue [65] |
|  | miR200-\|Zeb1 | Human breast cancer cell line, NCI-60 cell lines [54,66] |
|  | NICD -> Zeb1 | Human pancreatic cancer cell line [67] |
| ZEB2 | Snai1 -> Zeb2 expression via alternative processing of its mRNA | Intestinal cancer cell line [68] |
|  | Twist1 & Snai2 cooperate to activate transcription of Zeb2 | Mammary epithelial cells [43] |
|  | miR200 -\| Zeb2 | Human breast cancer cell line, NCI-60 cell lines [54,66] |
|  | miR203-\|Zeb2 | Human breast cancer cell line, human prostate cell lines [54,69] |
|  | NICD -> Zeb2 | Human pancreatic cancer cell line [67] |
| AKT1 | NICD -> AKT | lymphoblastic leukemia cell line [70] |
|  | CTNNB1 -> AKT1 | Human glioblastoma cell lines [71] |
|  | TP53 -\| AKT through transcription of PTEN which inhibits AKT activation | Madin-Darby canine kidney cells [72] |
|  | miR34 -\| AKT-P mir34 inhibits AKT activation | The human uveal melanoma cell line [73] |
|  | TGF-β -> AKT1 through p38 MapK | Lung Fibroblasts [74] |
|  | CDH2 can activate FGFR leading to activation of AKT1 | Mice [75] |
|  | E-cadherin inhibits ligand binding to EGFR at high cell densities | Breast and gastric cancers [76] |
| DKK1 | CTNNB1 -> DKK1 | Prostate, human embryonic kidney cells and human colon cancer [77,78] |
|  | NICD->DKK | Intestinal stem cells [79] |
|  | DKK1 is methylated in colon cancer | CRC tumours [80] |
| CTNNB1 | DKK1 -\| CTNNB1 | Colon cancer [81] |
|  | AKT1 ->Chibby -> CTNNB1-P in nucleus and this results in export of CTNNB1 into the cytosol | Colon cancer [82] |
|  | CDH1-\| Wnt-signalling by forming complex with b-catenin | epidermoid carcinoma, breast cancer cell line [83,84] |
|  | CHD2 -\| Wnt-signalling by forming also complexes with b-catenin | Myofibroblast [17] |
|  | miR34 -\| CTNNB1 | Mouse embryonic fibroblast, lung carcinoma and colon carcinoma cell line [85] |
|  | miR200 -\|CTNNB1 | Meningioma cell lines, nasopharyngeal carcinoma [86,87] |
|  | p63 -\| CTNNB1 via increased transcription of APC | Osteosarcoma [88] |
|  | p53 -\| CTNBB1 via activation of nuclear GSK3 -\| CTNNB1 | Lung cancer, neuroblastoma [89] |
| NICD | p53-\|PSEN->NICD | lymphoblastic leukemia cell line  [70] |
|  | p63 and p73->Jag->NICD Not included in this model because the model represents a single cell system. Therefore p63 and p73-\|NICD as it has been shown that cis-interaction between Jag ligands inhibit Notch signalling within the same cell | Drosophila S2 cells, mouse myoblast [90–93] |
|  | miR200 -\| NICD | Breast adenocarcinoma pancreatic adenocarcinoma [94] |
|  | miR34 -\| Notch1 = NICD | cervical carcinoma and choriocarcinoma cells [95] |
| p63 | Notch inhibits p63 | lymphoblastic leukemia cell line  [70,96,97] |
|  | AKT inhibits p63 via mdm2 | lymphoblastic leukemia cell line, *in vitro* [70,98] |
|  | p53 inhibits p63 via p53-PTEN-AKT-mdm2 | Transformed fibroblast cells [99,100] |
|  | miR203 -\| p63 | Myoblast, keratinocytes [101,102] |
| p53 | NICD -> p53 Not included in the model because cell type dependent | lymphoblastic leukemia cell line  [70] |
|  | p73 -\| 53 | human non-small cell lung carcinoma, human mammary adenocarcinoma, osteogenic sarcoma [103,104] |
|  | Snai2 inhibits p53 expression | Madin-Darby canine kidney cells [20] |
|  | AKT1 inhibits p53 via MDM2 | Prostate epithelial cells [105] |
|  | AKT2 inhibits p53 via MDMD2 | Prostate epithelial cells [105] |
|  | CTNNB1 -> p53 at protein level through inhibiting mdm2 and through p14ARF | lymph node metastatic cells, endometrial carcinoma cells [106,107] |
|  | NICD -> myc -> p14ARF->p53 | Fibroblast, T-acute lymphoblastic leukemia [35,108] |
|  | mir34a -\| sirt1 -\| p53 | Colon carcinoma [109] |
| p73 | p53 inhibit p73 expression | ovarian epithelium cells [110] |
|  | AKT1/2 inhibits p73 via mdm2 | lymphoblastic leukemia cell line, HeLa cells [70,111] |
|  | Zeb1 -\| p73 expression | Ovarian epithelium cells [110] |
| miR200 | p53 activates transcription of miR200c | Keratinocytes [45] |
|  | p63 and p73 activate transcription of miR200 family | human ovarian surface epithelial cells [112] |
|  | Zeb1 and Zeb2 inhibit expression of miR200 | Human breast cancer cell line, NCI-60 cell lines [54,66] |
|  | Snai1 and Snai2 inhibit expression of miR200 | Human breast cancer cell line [54] |
|  | AKT2 inhibits miR200 | Lung fibroblast and kidney epithelia cells [113,114] |
| miR203 | p53 activates transcription of miR203 in keratinocytes | Keratinocytes [115] |
|  | Snai1 inhibits expression of miR203 | human prostate cell lines [69] |
|  | Zeb1 and Zeb2 inhibit expression of miR203 | Human breast cancer cell line [54] |
| miR34 | miR34 is a p53 target gene | Mouse embryonic fibroblast, lung carcinoma, breast cancer, osteosarcoma and colon carcinoma cell line [85,116] |
|  | Snai1 and Zeb1 regulate miR34 expression | Colon adenocarcinoma [55] |
|  | p63 represses miR34 | Keratinocytes [117] |
|  | p73 induces gene transcription miR34 | Mouse cortical neurons [118] |
|  | AKT1 inhibits miR34 | Breast cancer [113] |
|  | AKT2 induces mir34 | Breast cancer [113] |
| AKT2 | Twist induces AKT2 transcription | Breast cancer cell line[3,4] |
|  | knock down of AKT1 increases AKT2 | Breast cancer [113] |
|  | miR203 inhibits AKT2 | Colon cancer [119] |
|  | miR34 inhibits AKT2 | Prostate cancer [120] |
|  | TGF-β activates PI3K through association of its receptors with p85 subunit | Epithelial cells [121] |
|  | CDH2 can activate FGFR thereby activating AKT2 | Mice [75] |
|  | p53 inhibits AKT2 through PTEN | [72] |
| ERK | TGF-β activates ERK | mammary adenocarcinoma, mouse fibroblast [10,122] |
|  | CDH2 can activates ERK via FGFR polymerisation | Mice [75] |
|  | AKT1 inhibits ERK signalling | Breast epithelial cells, breast cancer cells [8,123] |
|  | NICD -> HES1 -\| DUSP1/6 -\| Erk | murine non-small cell lung cancer [124] |
| SMAD | miR203 -\| smad4: transcriptional component of the TGF-b pathway | hepatocellular carcinoma [28] |
|  | miR200 -\|Smad2 and 3 | Intestinal epithelial cells [125] |
| TGF-β | CTNNB1 -> Bambi -\| TGF-β | Colorectal tumours [126] |
|  | Notch -> Nodal -> TGF-β | Heart tissue from zebrafish [127] |
|  |  |  |
| GF | CDH1 -\| GF  CDH2 -> GF  GF autoregulation: the cell itself can produce ligands and thus it can activate the receptor tyrosine kinase family of receptors and eventually stimulate the cell to produce GF. Cis-inhibition observed for Notch receptors has not been demonstrated for RTK | Ovarian cancer cells [128]  Melanoma cells.  In mice, ovarian carcinoma cells, rat intestinal epithelial cells , ovarian carcinoma cells [57,75,129]  *Drosophila melanogaster* [130] |
| ECMicroenv | The extracellular microenvironment refers to the extracellular matrix. ECMicroenv is an input of the model |  |
| DNAdamage | DNA damage refers to any insults that lead to activation of p53 family members. DNAdamage is an input of the model. |  |

### References:

1. Arboleda MJ, Lyons JF, Kabbinavar FF, Bray MR, Snow BE, et al. (2003) Overexpression of AKT2/protein kinase Bbeta leads to up-regulation of beta1 integrins, increased invasion, and metastasis of human breast and ovarian cancer cells. Cancer Res 63: 196–206.

2. Bulj Z, Duchi S, Bevilacqua A, Gherardi A, Dozza B, et al. (2013) Protein kinase B/AKT isoform 2 drives migration of human mesenchymal stem cells. Int J Oncol 42: 118–126. doi:10.3892/ijo.2012.1700.

3. Cheng GZ, Chan J, Wang Q, Zhang W, Sun CD, et al. (2007) Twist transcriptionally up-regulates AKT2 in breast cancer cells leading to increased migration, invasion, and resistance to paclitaxel. Cancer Res 67: 1979–1987. doi:10.1158/0008-5472.CAN-06-1479.

4. Cheng GZ, Zhang W, Wang L-H (2008) Regulation of cancer cell survival, migration, and invasion by Twist: AKT2 comes to interplay. Cancer Res 68: 957–960. doi:10.1158/0008-5472.CAN-07-5067.

5. Chin YR, Toker A (2010) The actin-bundling protein palladin is an Akt1-specific substrate that regulates breast cancer cell migration. Mol Cell 38: 333–344. doi:10.1016/j.molcel.2010.02.031.

6. Kimura H, Okubo N, Chosa N, Kyakumoto S, Kamo M, et al. (2013) EGF positively regulates the proliferation and migration, and negatively regulates the myofibroblast differentiation of periodontal ligament-derived endothelial progenitor cells through MEK/ERK- and JNK-dependent signals. Cell Physiol Biochem 32: 899–914. doi:10.1159/000354493.

7. Howe EN, Cochrane DR, Richer JK (2011) Targets of miR-200c mediate suppression of cell motility and anoikis resistance. Breast Cancer Res 13: R45. doi:10.1186/bcr2867.

8. Irie HY, Pearline R V, Grueneberg D, Hsia M, Ravichandran P, et al. (2005) Distinct roles of Akt1 and Akt2 in regulating cell migration and epithelial-mesenchymal transition. J Cell Biol 171: 1023–1034. doi:10.1083/jcb.200505087.

9. Kim E-S, Kim M-S, Moon A (2004) TGF-beta-induced upregulation of MMP-2 and MMP-9 depends on p38 MAPK, but not ERK signaling in MCF10A human breast epithelial cells. Int J Oncol 25: 1375–1382.

10. Daroqui MC, Vazquez P, Bal de Kier Joffé E, Bakin A V, Puricelli LI (2012) TGF-β autocrine pathway and MAPK signaling promote cell invasiveness and in vivo mammary adenocarcinoma tumor progression. Oncol Rep 28: 567–575. doi:10.3892/or.2012.1813.

11. Jean C, Blanc A, Prade-Houdellier N, Ysebaert L, Hernandez-Pigeon H, et al. (2009) Epidermal growth factor receptor/beta-catenin/T-cell factor 4/matrix metalloproteinase 1: a new pathway for regulating keratinocyte invasiveness after UVA irradiation. Cancer Res 69: 3291–3299. doi:10.1158/0008-5472.CAN-08-1909.

12. Planutiene M, Planutis K, Holcombe RF (2011) Lymphoid enhancer-binding factor 1, a representative of vertebrate-specific Lef1/Tcf1 sub-family, is a Wnt-beta-catenin pathway target gene in human endothelial cells which regulates matrix metalloproteinase-2 expression and promotes endothelial cell inva. Vasc Cell 3: 28. doi:10.1186/2045-824X-3-28.

13. Blavier L, Lazaryev A, Shi X-H, Dorey FJ, Shackleford GM, et al. (2010) Stromelysin-1 (MMP-3) is a target and a regulator of Wnt1-induced epithelial-mesenchymal transition (EMT). Cancer Biol Ther 10: 198–208.

14. Gustavson MD, Crawford HC, Fingleton B, Matrisian LM (2004) Tcf binding sequence and position determines beta-catenin and Lef-1 responsiveness of MMP-7 promoters. Mol Carcinog 41: 125–139. doi:10.1002/mc.20049.

15. Wu B, Crampton SP, Hughes CCW (2007) Wnt signaling induces matrix metalloproteinase expression and regulates T cell transmigration. Immunity 26: 227–239. doi:10.1016/j.immuni.2006.12.007.

16. Yun K, Im S-H (2007) Transcriptional regulation of MMP13 by Lef1 in chondrocytes. Biochem Biophys Res Commun 364: 1009–1014. doi:10.1016/j.bbrc.2007.10.121.

17. De Wever O, Westbroek W, Verloes A, Bloemen N, Bracke M, et al. (2004) Critical role of N-cadherin in myofibroblast invasion and migration in vitro stimulated by colon-cancer-cell-derived TGF-beta or wounding. J Cell Sci 117: 4691–4703. doi:10.1242/jcs.01322.

18. Futterman MA, García AJ, Zamir EA (2011) Evidence for partial epithelial-to-mesenchymal transition (pEMT) and recruitment of motile blastoderm edge cells during avian epiboly. Dev Dyn 240: 1502–1511. doi:10.1002/dvdy.22607.

19. Nieto MA (2011) The ins and outs of the epithelial to mesenchymal transition in health and disease. Annu Rev Cell Dev Biol 27: 347–376. doi:10.1146/annurev-cellbio-092910-154036.

20. Leroy P, Mostov KE (2007) Slug is required for cell survival during partial epithelial-mesenchymal transition of HGF-induced tubulogenesis. Mol Biol Cell 18: 1943–1952. doi:10.1091/mbc.E06-09-0823.

21. Akaogi K, Ono W, Hayashi Y, Kishimoto H, Yanagisawa J (2013) MYBBP1A suppresses breast cancer tumorigenesis by enhancing the p53 dependent anoikis. BMC Cancer 13: 65. doi:10.1186/1471-2407-13-65.

22. Dufour G, Demers M-J, Gagné D, Dydensborg AB, Teller IC, et al. (2004) Human intestinal epithelial cell survival and anoikis. Differentiation state-distinct regulation and roles of protein kinase B/Akt isoforms. J Biol Chem 279: 44113–44122. doi:10.1074/jbc.M405323200.

23. Carroll DK, Carroll JS, Leong C-O, Cheng F, Brown M, et al. (2006) p63 regulates an adhesion programme and cell survival in epithelial cells. Nat Cell Biol 8: 551–561. doi:10.1038/ncb1420.

24. De Krijger I, Mekenkamp LJM, Punt CJA, Nagtegaal ID (2011) MicroRNAs in colorectal cancer metastasis. J Pathol 224: 438–447. doi:10.1002/path.2922.

25. Zhu W, Xu H, Zhu D, Zhi H, Wang T, et al. (2012) miR-200bc/429 cluster modulates multidrug resistance of human cancer cell lines by targeting BCL2 and XIAP. Cancer Chemother Pharmacol 69: 723–731. doi:10.1007/s00280-011-1752-3.

26. Neuzillet C, Hammel P, Tijeras-Raballand A, Couvelard A, Raymond E (2013) Targeting the Ras-ERK pathway in pancreatic adenocarcinoma. Cancer Metastasis Rev 32: 147–162. doi:10.1007/s10555-012-9396-2.

27. Lee Y-M, Lee J-Y, Ho C-C, Hong Q-S, Yu S-L, et al. (2011) miRNA-34b as a tumor suppressor in estrogen-dependent growth of breast cancer cells. Breast Cancer Res 13: R116. doi:10.1186/bcr3059.

28. Furuta M, Kozaki K, Tanaka S, Arii S, Imoto I, et al. (2010) miR-124 and miR-203 are epigenetically silenced tumor-suppressive microRNAs in hepatocellular carcinoma. Carcinogenesis 31: 766–776. doi:10.1093/carcin/bgp250.

29. Cai J, Liu X, Cheng J, Li Y, Huang X, et al. (2012) MicroRNA-200 is commonly repressed in conjunctival MALT lymphoma, and targets cyclin E2. Graefes Arch Clin Exp Ophthalmol 250: 523–531. doi:10.1007/s00417-011-1885-4.

30. Héron-Milhavet L, Franckhauser C, Rana V, Berthenet C, Fisher D, et al. (2006) Only Akt1 is required for proliferation, while Akt2 promotes cell cycle exit through p21 binding. Mol Cell Biol 26: 8267–8280. doi:10.1128/MCB.00201-06.

31. Sayan AE, Griffiths TR, Pal R, Browne GJ, Ruddick A, et al. (2009) SIP1 protein protects cells from DNA damage-induced apoptosis and has independent prognostic value in bladder cancer. Proc Natl Acad Sci U S A 106: 14884–14889. doi:10.1073/pnas.0902042106.

32. Murray-Zmijewski F, Lane DP, Bourdon J-C (2006) p53/p63/p73 isoforms: an orchestra of isoforms to harmonise cell differentiation and response to stress. Cell Death Differ 13: 962–972. doi:10.1038/sj.cdd.4401914.

33. Dohn M, Zhang S, Chen X (2001) p63alpha and DeltaNp63alpha can induce cell cycle arrest and apoptosis and differentially regulate p53 target genes. Oncogene 20: 3193–3205. doi:10.1038/sj.onc.1204427.

34. Allocati N, Di Ilio C, De Laurenzi V (2012) p63/p73 in the control of cell cycle and cell death. Exp Cell Res 318: 1285–1290. doi:10.1016/j.yexcr.2012.01.023.

35. Harris SL, Levine AJ (2005) The p53 pathway: positive and negative feedback loops. Oncogene 24: 2899–2908. doi:10.1038/sj.onc.1208615.

36. Agarwal ML, Agarwal A, Taylor WR, Stark GR (1995) p53 controls both the G2/M and the G1 cell cycle checkpoints and mediates reversible growth arrest in human fibroblasts. Proc Natl Acad Sci U S A 92: 8493–8497.

37. Niimi H, Pardali K, Vanlandewijck M, Heldin C-H, Moustakas A (2007) Notch signaling is necessary for epithelial growth arrest by TGF-beta. J Cell Biol 176: 695–707. doi:10.1083/jcb.200612129.

38. Takahashi E, Funato N, Higashihori N, Hata Y, Gridley T, et al. (2004) Snail regulates p21(WAF/CIP1) expression in cooperation with E2A and Twist. Biochem Biophys Res Commun 325: 1136–1144. doi:10.1016/j.bbrc.2004.10.148.

39. Heron-Milhavet L, Franckhauser C, Fernandez A, Lamb NJ (2013) Characterization of the Akt2 domain essential for binding nuclear p21cip1 to promote cell cycle arrest during myogenic differentiation. PLoS One 8: e76987. doi:10.1371/journal.pone.0076987.

40. Villagrasa P, Díaz VM, Viñas-Castells R, Peiró S, Del Valle-Pérez B, et al. (2012) Akt2 interacts with Snail1 in the E-cadherin promoter. Oncogene 31: 4022–4033. doi:10.1038/onc.2011.562.

41. Le Bras GF, Taubenslag KJ, Andl CD (2012) The regulation of cell-cell adhesion during epithelial-mesenchymal transition, motility and tumor progression. Cell Adh Migr 6: 365–373. doi:10.4161/cam.21326.

42. Wels C, Joshi S, Koefinger P, Bergler H, Schaider H (2011) Transcriptional activation of ZEB1 by Slug leads to cooperative regulation of the epithelial-mesenchymal transition-like phenotype in melanoma. J Invest Dermatol 131: 1877–1885. doi:10.1038/jid.2011.142.

43. Casas E, Kim J, Bendesky A, Ohno-Machado L, Wolfe CJ, et al. (2011) Snail2 is an essential mediator of Twist1-induced epithelial mesenchymal transition and metastasis. Cancer Res 71: 245–254. doi:10.1158/0008-5472.CAN-10-2330.

44. Sánchez-Tilló E, Liu Y, de Barrios O, Siles L, Fanlo L, et al. (2012) EMT-activating transcription factors in cancer: beyond EMT and tumor invasiveness. Cell Mol Life Sci 69: 3429–3456. doi:10.1007/s00018-012-1122-2.

45. Hao L, Ha JR, Kuzel P, Garcia E, Persad S (2012) Cadherin switch from E- to N-cadherin in melanoma progression is regulated by the PI3K/PTEN pathway through Twist and Snail. Br J Dermatol 166: 1184–1197. doi:10.1111/j.1365-2133.2012.10824.x.

46. Gilles C, Polette M, Mestdagt M, Nawrocki-Raby B, Ruggeri P, et al. (2003) Transactivation of vimentin by beta-catenin in human breast cancer cells. Cancer Res 63: 2658–2664.

47. Yates B, Zetterberg C, Rajeev V, Reiss M, Rittling SR (2007) Promoter-independent regulation of vimentin expression in mammary epithelial cells by val(12)ras and TGFbeta. Exp Cell Res 313: 3718–3728. doi:10.1016/j.yexcr.2007.07.026.

48. Okugawa Y, Inoue Y, Tanaka K, Kawamura M, Saigusa S, et al. (2013) Smad interacting protein 1 (SIP1) is associated with peritoneal carcinomatosis in intestinal type gastric cancer. Clin Exp Metastasis 30: 417–429. doi:10.1007/s10585-012-9547-4.

49. Tran DD, Corsa CAS, Biswas H, Aft RL, Longmore GD (2011) Temporal and spatial cooperation of Snail1 and Twist1 during epithelial-mesenchymal transition predicts for human breast cancer recurrence. Mol Cancer Res 9: 1644–1657. doi:10.1158/1541-7786.MCR-11-0371.

50. Hsu K-W, Hsieh R-H, Huang K-H, Fen-Yau Li A, Chi C-W, et al. (2012) Activation of the Notch1/STAT3/Twist signaling axis promotes gastric cancer progression. Carcinogenesis 33: 1459–1467. doi:10.1093/carcin/bgs165.

51. Heuberger J, Birchmeier W (2010) Interplay of cadherin-mediated cell adhesion and canonical Wnt signaling. Cold Spring Harb Perspect Biol 2: a002915. doi:10.1101/cshperspect.a002915.

52. Dave N, Guaita-Esteruelas S, Gutarra S, Frias À, Beltran M, et al. (2011) Functional cooperation between Snail1 and twist in the regulation of ZEB1 expression during epithelial to mesenchymal transition. J Biol Chem 286: 12024–12032. doi:10.1074/jbc.M110.168625.

53. Sahlgren C, Gustafsson M V, Jin S, Poellinger L, Lendahl U (2008) Notch signaling mediates hypoxia-induced tumor cell migration and invasion. Proc Natl Acad Sci U S A 105: 6392–6397. doi:10.1073/pnas.0802047105.

54. Moes M, Le Béchec A, Crespo I, Laurini C, Halavatyi A, et al. (2012) A Novel Network Integrating a miRNA-203/SNAI1 Feedback Loop which Regulates Epithelial to Mesenchymal Transition. PLoS One 7: e35440. doi:10.1371/journal.pone.0035440.

55. Siemens H, Jackstadt R, Hünten S, Kaller M, Menssen A, et al. (2011) miR-34 and SNAIL form a double-negative feedback loop to regulate epithelial-mesenchymal transitions. Cell Cycle 10: 4256–4271. doi:10.4161/cc.10.24.18552.

56. Lim S-O, Kim H, Jung G (2010) p53 inhibits tumor cell invasion via the degradation of snail protein in hepatocellular carcinoma. FEBS Lett 584: 2231–2236. doi:10.1016/j.febslet.2010.04.006.

57. Liao Y, Lönnerdal B (2010) Beta-catenin/TCF4 transactivates miR-30e during intestinal cell differentiation. Cell Mol Life Sci 67: 2969–2978. doi:10.1007/s00018-010-0366-y.

58. Kumarswamy R, Mudduluru G, Ceppi P, Muppala S, Kozlowski M, et al. (2012) MicroRNA-30a inhibits epithelial-to-mesenchymal transition by targeting Snai1 and is downregulated in non-small cell lung cancer. Int J Cancer 130: 2044–2053. doi:10.1002/ijc.26218.

59. Sakai D, Tanaka Y, Endo Y, Osumi N, Okamoto H, et al. (2005) Regulation of Slug transcription in embryonic ectoderm by beta-catenin-Lef/Tcf and BMP-Smad signaling. Dev Growth Differ 47: 471–482. doi:10.1111/j.1440-169X.2005.00821.x.

60. Leong KG, Niessen K, Kulic I, Raouf A, Eaves C, et al. (2007) Jagged1-mediated Notch activation induces epithelial-to-mesenchymal transition through Slug-induced repression of E-cadherin. J Exp Med 204: 2935–2948. doi:10.1084/jem.20071082.

61. Niessen K, Fu Y, Chang L, Hoodless PA, McFadden D, et al. (2008) Slug is a direct Notch target required for initiation of cardiac cushion cellularization. J Cell Biol 182: 315–325. doi:10.1083/jcb.200710067.

62. Liu Y-N, Yin JJ, Abou-Kheir W, Hynes PG, Casey OM, et al. (2012) MiR-1 and miR-200 inhibit EMT via Slug-dependent and tumorigenesis via Slug-independent mechanisms. Oncogene. doi:10.1038/onc.2012.58.

63. Wang S-P, Wang W-L, Chang Y-L, Wu C-T, Chao Y-C, et al. (2009) p53 controls cancer cell invasion by inducing the MDM2-mediated degradation of Slug. Nat Cell Biol 11: 694–704. doi:10.1038/ncb1875.

64. Qu Y, Li W-C, Hellem MR, Rostad K, Popa M, et al. (2013) MiR-182 and miR-203 induce mesenchymal to epithelial transition and self-sufficiency of growth signals via repressing SNAI2 in prostate cells. Int J Cancer 133: 544–555. doi:10.1002/ijc.28056.

65. Sánchez-Tilló E, de Barrios O, Siles L, Cuatrecasas M, Castells A, et al. (2011) β-catenin/TCF4 complex induces the epithelial-to-mesenchymal transition (EMT)-activator ZEB1 to regulate tumor invasiveness. Proc Natl Acad Sci U S A 108: 19204–19209. doi:10.1073/pnas.1108977108.

66. Hill L, Browne G, Tulchinsky E (2012) ZEB/miR-200 feedback loop: At the crossroads of signal transduction in cancer. Int J Cancer. doi:10.1002/ijc.27708.

67. Bao B, Wang Z, Ali S, Kong D, Li Y, et al. (2011) Notch-1 induces epithelial-mesenchymal transition consistent with cancer stem cell phenotype in pancreatic cancer cells. Cancer Lett 307: 26–36. doi:10.1016/j.canlet.2011.03.012.

68. Beltran M, Puig I, Peña C, García JM, Alvarez AB, et al. (2008) A natural antisense transcript regulates Zeb2/Sip1 gene expression during Snail1-induced epithelial-mesenchymal transition. Genes Dev 22: 756–769. doi:10.1101/gad.455708.

69. Saini S, Majid S, Yamamura S, Tabatabai L, Suh SO, et al. (2011) Regulatory Role of mir-203 in Prostate Cancer Progression and Metastasis. Clin Cancer Res 17: 5287–5298. doi:10.1158/1078-0432.CCR-10-2619.

70. Dotto GP (2009) Crosstalk of Notch with p53 and p63 in cancer growth control. Nat Rev Cancer 9: 587–595. doi:10.1038/nrc2675.

71. Chen L, Huang K, Han L, Shi Z, Zhang K, et al. (2011) β-catenin/Tcf-4 complex transcriptionally regulates AKT1 in glioma. Int J Oncol 39: 883–890. doi:10.3892/ijo.2011.1104.

72. Escrivà M, Peiró S, Herranz N, Villagrasa P, Dave N, et al. (2008) Repression of PTEN phosphatase by Snail1 transcriptional factor during gamma radiation-induced apoptosis. Mol Cell Biol 28: 1528–1540. doi:10.1128/MCB.02061-07.

73. Dong F, Lou D (2012) MicroRNA-34b/c suppresses uveal melanoma cell proliferation and migration through multiple targets. Mol Vis 18: 537–546.

74. Horowitz JC, Lee DY, Waghray M, Keshamouni VG, Thomas PE, et al. (2004) Activation of the pro-survival phosphatidylinositol 3-kinase/AKT pathway by transforming growth factor-beta1 in mesenchymal cells is mediated by p38 MAPK-dependent induction of an autocrine growth factor. J Biol Chem 279: 1359–1367. doi:10.1074/jbc.M306248200.

75. Qian X, Anzovino A, Kim S, Suyama K, Yao J, et al. (2013) N-cadherin/FGFR promotes metastasis through epithelial-to-mesenchymal transition and stem/progenitor cell-like properties. Oncogene. doi:10.1038/onc.2013.310.

76. Van Roy F, Berx G (2008) The cell-cell adhesion molecule E-cadherin. Cell Mol Life Sci 65: 3756–3788. doi:10.1007/s00018-008-8281-1.

77. Niida A, Hiroko T, Kasai M, Furukawa Y, Nakamura Y, et al. (2004) DKK1, a negative regulator of Wnt signaling, is a target of the beta-catenin/TCF pathway. Oncogene 23: 8520–8526. doi:10.1038/sj.onc.1207892.

78. Pendás-Franco N, García JM, Peña C, Valle N, Pálmer HG, et al. (2008) DICKKOPF-4 is induced by TCF/beta-catenin and upregulated in human colon cancer, promotes tumour cell invasion and angiogenesis and is repressed by 1alpha,25-dihydroxyvitamin D3. Oncogene 27: 4467–4477. doi:10.1038/onc.2008.88.

79. Katoh M, Katoh M (2007) WNT antagonist, DKK2, is a Notch signaling target in intestinal stem cells: augmentation of a negative regulation system for canonical WNT signaling pathway by the Notch-DKK2 signaling loop in primates. Int J Mol Med 19: 197–201.

80. Menezes ME, Devine DJ, Shevde LA, Samant RS (2012) Dickkopf1: a tumor suppressor or metastasis promoter? Int J Cancer 130: 1477–1483. doi:10.1002/ijc.26449.

81. González-Sancho JM, Aguilera O, García JM, Pendás-Franco N, Peña C, et al. (2005) The Wnt antagonist DICKKOPF-1 gene is a downstream target of beta-catenin/TCF and is downregulated in human colon cancer. Oncogene 24: 1098–1103. doi:10.1038/sj.onc.1208303.

82. Takemaru K-I, Fischer V, Li F-Q (2009) Fine-tuning of nuclear-catenin by Chibby and 14-3-3. Cell Cycle 8: 210–213.

83. Tian X, Liu Z, Niu B, Zhang J, Tan TK, et al. (2011) E-cadherin/β-catenin complex and the epithelial barrier. J Biomed Biotechnol 2011: 567305. doi:10.1155/2011/567305.

84. Ma L, Young J, Prabhala H, Pan E, Mestdagh P, et al. (2010) miR-9, a MYC/MYCN-activated microRNA, regulates E-cadherin and cancer metastasis. Nat Cell Biol 12: 247–256. doi:10.1038/ncb2024.

85. Kim NH, Kim HS, Li X-Y, Lee I, Choi H-S, et al. (2011) A p53/miRNA-34 axis regulates Snail1-dependent cancer cell epithelial-mesenchymal transition. J Cell Biol 195: 417–433. doi:10.1083/jcb.201103097.

86. Saydam O, Shen Y, Würdinger T, Senol O, Boke E, et al. (2009) Downregulated microRNA-200a in meningiomas promotes tumor growth by reducing E-cadherin and activating the Wnt/beta-catenin signaling pathway. Mol Cell Biol 29: 5923–5940. doi:10.1128/MCB.00332-09.

87. Xia H, Ng SS, Jiang S, Cheung WKC, Sze J, et al. (2010) miR-200a-mediated downregulation of ZEB2 and CTNNB1 differentially inhibits nasopharyngeal carcinoma cell growth, migration and invasion. Biochem Biophys Res Commun 391: 535–541. doi:10.1016/j.bbrc.2009.11.093.

88. Wu G, Nomoto S, Hoque MO, Dracheva T, Osada M, et al. (2003) DeltaNp63alpha and TAp63alpha regulate transcription of genes with distinct biological functions in cancer and development. Cancer Res 63: 2351–2357.

89. Watcharasit P, Bijur GN, Zmijewski JW, Song L, Zmijewska A, et al. (2002) Direct, activating interaction between glycogen synthase kinase-3beta and p53 after DNA damage. Proc Natl Acad Sci U S A 99: 7951–7955. doi:10.1073/pnas.122062299.

90. Cordle J, Johnson S, Tay JZY, Roversi P, Wilkin MB, et al. (2008) A conserved face of the Jagged/Serrate DSL domain is involved in Notch trans-activation and cis-inhibition. Nat Struct Mol Biol 15: 849–857. doi:10.1038/nsmb.1457.

91. Del Álamo D, Rouault H, Schweisguth F (2011) Mechanism and significance of cis-inhibition in Notch signalling. Curr Biol 21: R40–R47. doi:10.1016/j.cub.2010.10.034.

92. Micchelli CA, Rulifson EJ, Blair SS (1997) The function and regulation of cut expression on the wing margin of Drosophila: Notch, Wingless and a dominant negative role for Delta and Serrate. Development 124: 1485–1495.

93. D’Souza B, Miyamoto A, Weinmaster G (2008) The many facets of Notch ligands. Oncogene 27: 5148–5167. doi:10.1038/onc.2008.229.

94. Brabletz S, Bajdak K, Meidhof S, Burk U, Niedermann G, et al. (2011) The ZEB1/miR-200 feedback loop controls Notch signalling in cancer cells. EMBO J 30: 770–782. doi:10.1038/emboj.2010.349.

95. Pang RTK, Leung CON, Ye T-M, Liu W, Chiu PCN, et al. (2010) MicroRNA-34a suppresses invasion through downregulation of Notch1 and Jagged1 in cervical carcinoma and choriocarcinoma cells. Carcinogenesis 31: 1037–1044. doi:10.1093/carcin/bgq066.

96. Forster N, Ellisen LW (2011) Notch signaling mediates p63-induced quiescence: a new facet of p63/Notch crosstalk. Cell Cycle 10: 3632–3633. doi:10.4161/cc.10.21.18182.

97. Roemer K (2012) Notch and the p53 clan of transcription factors. Adv Exp Med Biol 727: 223–240. doi:10.1007/978-1-4614-0899-4_17.

98. Zdzalik M, Pustelny K, Kedracka-Krok S, Huben K, Pecak A, et al. (2010) Interaction of regulators Mdm2 and Mdmx with transcription factors p53, p63 and p73. Cell Cycle 9: 4584–4591.

99. Feng Z, Levine AJ (2010) The regulation of energy metabolism and the IGF-1/mTOR pathways by the p53 protein. Trends Cell Biol 20: 427–434. doi:10.1016/j.tcb.2010.03.004.

100. Moll UM, Petrenko O (2003) The MDM2-p53 interaction. Mol Cancer Res 1: 1001–1008.

101. Murakami M, Ohkuma M, Nakamura M (2008) Molecular mechanism of transforming growth factor-beta-mediated inhibition of growth arrest and differentiation in a myoblast cell line. Dev Growth Differ 50: 121–130. doi:10.1111/j.1440-169X.2007.00982.x.

102. Lena AM, Shalom-Feuerstein R, Rivetti di Val Cervo P, Aberdam D, Knight RA, et al. (2008) miR-203 represses “stemness” by repressing DeltaNp63. Cell Death Differ 15: 1187–1195. doi:10.1038/cdd.2008.69.

103. Bailey SG, Cragg MS, Townsend PA (2011) Family friction as ΔNp73 antagonises p73 and p53. Int J Biochem Cell Biol 43: 482–486. doi:10.1016/j.biocel.2010.12.022.

104. Wang XQ, Ongkeko WM, Lau AW, Leung KM, Poon RY (2001) A possible role of p73 on the modulation of p53 level through MDM2. Cancer Res 61: 1598–1603.

105. Moro L, Arbini AA, Yao JL, di Sant’Agnese PA, Marra E, et al. (2009) Mitochondrial DNA depletion in prostate epithelial cells promotes anoikis resistance and invasion through activation of PI3K/Akt2. Cell Death Differ 16: 571–583. doi:10.1038/cdd.2008.178.

106. Damalas A, Ben-Ze’ev A, Simcha I, Shtutman M, Leal JF, et al. (1999) Excess beta-catenin promotes accumulation of transcriptionally active p53. EMBO J 18: 3054–3063. doi:10.1093/emboj/18.11.3054.

107. Saegusa M, Hashimura M, Kuwata T, Hamano M, Okayasu I (2004) Beta-catenin simultaneously induces activation of the p53-p21WAF1 pathway and overexpression of cyclin D1 during squamous differentiation of endometrial carcinoma cells. Am J Pathol 164: 1739–1749.

108. Aster JC, Bodnar N, Xu L, Karnell F, Milholland JM, et al. (2011) Notch ankyrin repeat domain variation influences leukemogenesis and Myc transactivation. PLoS One 6: e25645. doi:10.1371/journal.pone.0025645.

109. Feng Z, Zhang C, Wu R, Hu W (2011) Tumor suppressor p53 meets microRNAs. J Mol Cell Biol 3: 44–50. doi:10.1093/jmcb/mjq040.

110. Blandino G, Dobbelstein M (2004) p73 and p63: why do we still need them? Cell Cycle 3: 886–894.

111. Kubo N, Okoshi R, Nakashima K, Shimozato O, Nakagawara A, et al. (2010) MDM2 promotes the proteasomal degradation of p73 through the interaction with Itch in HeLa cells. Biochem Biophys Res Commun 403: 405–411. doi:10.1016/j.bbrc.2010.11.043.

112. Knouf EC, Garg K, Arroyo JD, Correa Y, Sarkar D, et al. (2012) An integrative genomic approach identifies p73 and p63 as activators of miR-200 microRNA family transcription. Nucleic Acids Res 40: 499–510. doi:10.1093/nar/gkr731.

113. Iliopoulos D, Polytarchou C, Hatziapostolou M, Kottakis F, Maroulakou IG, et al. (2009) MicroRNAs differentially regulated by Akt isoforms control EMT and stem cell renewal in cancer cells. Sci Signal 2: ra62. doi:10.1126/scisignal.2000356.

114. Shimizu K, Chiba S, Saito T, Kumano K, Hamada Y, et al. (2002) Functional diversity among Notch1, Notch2, and Notch3 receptors. Biochem Biophys Res Commun 291: 775–779. doi:10.1006/bbrc.2002.6528.

115. McKenna DJ, McDade SS, Patel D, McCance DJ (2010) MicroRNA 203 expression in keratinocytes is dependent on regulation of p53 levels by E6. J Virol 84: 10644–10652. doi:10.1128/JVI.00703-10.

116. Tarasov V, Jung P, Verdoodt B, Lodygin D, Epanchintsev A, et al. (2007) Differential regulation of microRNAs by p53 revealed by massively parallel sequencing: miR-34a is a p53 target that induces apoptosis and G1-arrest. Cell Cycle 6: 1586–1593.

117. Antonini D, Russo MT, De Rosa L, Gorrese M, Del Vecchio L, et al. (2010) Transcriptional repression of miR-34 family contributes to p63-mediated cell cycle progression in epidermal cells. J Invest Dermatol 130: 1249–1257. doi:10.1038/jid.2009.438.

118. Agostini M, Tucci P, Steinert JR, Shalom-Feuerstein R, Rouleau M, et al. (2011) microRNA-34a regulates neurite outgrowth, spinal morphology, and function. Proc Natl Acad Sci U S A 108: 21099–21104. doi:10.1073/pnas.1112063108.

119. Li J, Chen Y, Zhao J, Kong F, Zhang Y (2011) miR-203 reverses chemoresistance in p53-mutated colon cancer cells through downregulation of Akt2 expression. Cancer Lett 304: 52–59. doi:10.1016/j.canlet.2011.02.003.

120. Majid S, Dar AA, Saini S, Shahryari V, Arora S, et al. (2013) miRNA-34b inhibits prostate cancer through demethylation, active chromatin modifications, and AKT pathways. Clin Cancer Res 19: 73–84. doi:10.1158/1078-0432.CCR-12-2952.

121. Yi JY, Shin I, Arteaga CL (2005) Type I transforming growth factor beta receptor binds to and activates phosphatidylinositol 3-kinase. J Biol Chem 280: 10870–10876. doi:10.1074/jbc.M413223200.

122. Hough C, Radu M, Doré JJE (2012) Tgf-beta induced Erk phosphorylation of smad linker region regulates smad signaling. PLoS One 7: e42513. doi:10.1371/journal.pone.0042513.

123. Toker A, Yoeli-Lerner M (2006) Akt signaling and cancer: surviving but not moving on. Cancer Res 66: 3963–3966. doi:10.1158/0008-5472.CAN-06-0743.

124. Baumgart A, Mazur PK, Anton M, Rudelius M, Schwamborn K, et al. (2014) Opposing role of Notch1 and Notch2 in a Kras(G12D)-driven murine non-small cell lung cancer model. Oncogene. doi:10.1038/onc.2013.592.

125. Chen Y, Xiao Y, Ge W, Zhou K, Wen J, et al. (2013) miR-200b inhibits TGF-β1-induced epithelial-mesenchymal transition and promotes growth of intestinal epithelial cells. Cell Death Dis 4: e541. doi:10.1038/cddis.2013.22.

126. Sekiya T, Adachi S, Kohu K, Yamada T, Higuchi O, et al. (2004) Identification of BMP and activin membrane-bound inhibitor (BAMBI), an inhibitor of transforming growth factor-beta signaling, as a target of the beta-catenin pathway in colorectal tumor cells. J Biol Chem 279: 6840–6846. doi:10.1074/jbc.M310876200.

127. Raya A, Koth CM, Büscher D, Kawakami Y, Itoh T, et al. (2003) Activation of Notch signaling pathway precedes heart regeneration in zebrafish. Proc Natl Acad Sci U S A 100 Suppl: 11889–11895. doi:10.1073/pnas.1834204100.

128. Lau M-T, Klausen C, Leung PCK (2011) E-cadherin inhibits tumor cell growth by suppressing PI3K/Akt signaling via β-catenin-Egr1-mediated PTEN expression. Oncogene 30: 2753–2766. doi:10.1038/onc.2011.6.

129. Talapatra S, Thompson CB (2001) Growth factor signaling in cell survival: implications for cancer treatment. J Pharmacol Exp Ther 298: 873–878.

130. VanHook AM (2013) Serrate-Mediated Cis-Inhibition. Sci Signal 6: ec90–ec90. doi:10.1126/scisignal.2004263.
