# Supplementary material for: Mathematical Modelling of Molecular Pathways Enabling Tumour Cell Invasion and Migration
Source: PLoS Comput Biol. 2015 Nov 3;11(11):e1004571. doi: 10.1371/journal.pcbi.1004571 (PMC4631357; doi:10.1371/journal.pcbi.1004571)
Supplement: S4 Table — For each condition or mutation, all possible inputs are considered. Thus, all possible outputs corresponding to stable states are shown in this table (values for internal variables are not shown). The existence of a stable state in accordance with what has been published is enough to conclude that the mutant is validated: there exists a condition for which the model explains the experiments. The fact that other stable states exist shows that for some particular conditions, the stable state could be reachable. For instance, for NICD GoF, we see that a stable state with metastasis exits which has not been observed in experiments. However, for this stable state, all p53 family members are OFF, thus, it is a particular situation. (DOCX) [file pcbi.1004571.s014.docx]

# Wild type

The table below shows the 9 stable states of the model obtained with GINsim. There are no limit cycles in the wild type condition (confirmed with MaBoSS).

The 9 stable states of the wild type correspond to all possible asymptotic solutions from all possible inputs (2^2^) where one input can have several corresponding stable states (the model is non-deterministic). For instance, if **ECMicroenv** is ON and **DNAdamage** is ON, it can lead to either **Apoptosis** (ss8) or **Metastasis** (ss9, which corresponds to a situation when all p53 family members are OFF). The stable states of the wild type represent all the possible solutions that the model allows without setting specific initial conditions (constraints).

The 9 stable states can be simplified into four families of phenotypes:

1. **HS** (ss1): with Cdh1 only and no output from the model. This stable state is considered as a homeostatic state where all inputs are OFF.

1. **Apoptosis + CellCycleArrest** (CCA) (ss3, ss4, ss7 and ss8): In these stable states, at least one of the TP53 family members is ON. For all cases, DNA damage is ON.

1. **EMT + CCA** (ss2 and ss5): For both stable states, TGFbeta and NICD are OFF. ECMicroenv is always OFF and DNA damage can be ON or OFF.

1. **Metastasis + Migration + Invasion + EMT + CCA** (ss6 and ss9): TGFbeta and NICD are ON, and the TP53 family members are all OFF. ECMicroenv is ON and DNA damage can be OFF or ON.

To help the readability of the results, we choose a simplified representation of the stable states that does not show explicitly show the inputs and the values of the other internal variables, but concentrates on the possible reachable phenotypes. Phenotypes are variables that we chose as outputs of the model:

|  | Apoptosis | CCA | EMT | Invasion | Migration | Metastasis | HS | Equivalent to: |
| --- | --- | --- | --- | --- | --- | --- | --- | --- |
| Wild type |  |  |  |  |  |  | \|  \| \| --- \| \|  \| 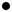 \|  \| \|  \|  \|  \| | ss1 |
|  | \|  \| \| --- \| \|  \| 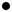 \|  \| \|  \|  \|  \| | \|  \| \| --- \| \|  \| 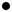 \|  \| \|  \|  \|  \| |  |  |  |  |  | ss3,ss4,ss7,ss8 |
|  |  | \|  \| \| --- \| \|  \| 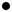 \|  \| \|  \|  \|  \| | \|  \| \| --- \| \|  \| 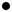 \|  \| \|  \|  \|  \| |  |  |  |  | ss2, ss5 |
|  |  | \|  \| \| --- \| \|  \| 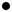 \|  \| \|  \|  \|  \| | \|  \| \| --- \| \|  \| 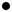 \|  \| \|  \|  \|  \| | \|  \| \| --- \| \|  \| 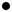 \|  \| \|  \|  \|  \| | \|  \| \| --- \| \|  \| 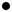 \|  \| \|  \|  \|  \| | \|  \| \| --- \| \|  \| 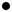 \|  \| \|  \|  \|  \| |  | ss6, ss9 |

Black circles are equivalent to a 1, and empty boxes are equivalent to a 0 in the table showing the 9 stable states above.

# Mutants

To simulate mutants, we force a variable to a value corresponding to the type of alterations: for GoF, the variable indicated in the column “Model variables” is set to the value 1, and for LoF, the variable is set to 0.

In the table below, we chose the simplified representation of the stable states, and thus we do not show the values of the inputs and of the internal variables. For more information about specific mutant, the model (SuppMat_Master_Model.zginml) can be simulated with GINsim.

We conclude that the results of the model are in accordance with the experimental results when one of the stable states is able to reproduce the description provided in the article. It means that there exists a condition in the model that fits the experiments.

An example for concluding that a mutant is in accordance with published experiments is given with TGF-β GoF: there exist 3 ‘families’ of stable states that lead to 3 phenotypes: metastasis, apoptosis and HS. Published experiments (reference 5) show that metastasis occurs when TGFbeta is added to the cell culture medium. We simulate this in our logical model by forcing TGFbeta into its active state: the logical rule for TGFbeta states that TGFbeta is always ON no matter the activity of its inputs and TGFbeta initial condition is set to 1. We confirm that there exists a particular combination of initial conditions that can lead to metastasis. The other stable states corresponding to an apoptotic phenotype or to a HS phenotype occur when GF (growth factors) are OFF, which could correspond to a particular condition met in the centre of the tumour.

| Mutation | Model variables | Apoptosis | CCA | EMT | Invasion | Migration | Metastasis | HS |  | Experimental results | Experimental model | Ref. | Comments for model results | limitations |
| --- | --- | --- | --- | --- | --- | --- | --- | --- | --- | --- | --- | --- | --- | --- |
| CTNNB1 GoF | CTNNB1=1 |  |  |  |  |  |  |  |  | adenoma, adenocarcinoma, infiltration | Mouse  model | [1] | With the model, we can say that CTNNB1 is involved in tumour initiation, EMT, and invasion but not migration because of AKT1. Thus, the model predicts that the PI3K/AKT pathway should be downregulated to migrate to distant sites |  |
|  |  |  |  |  |  |  |  |  |  |  |  |  |  |  |
| p53 LoF  Cdh1 LoF | p53=0  Cdh1=0 |  |  |  |  |  |  |  |  | Cdh1 -/- p53 -/- show accelerated tumours development and "distant" metastasis in lymph node | Mouse  model | [2] | The metastatic stable state is similar to the one of p53 LOF. Accelerated tumorigenesis cannot be shown with the model |  |
|  |  |  |  |  |  |  |  |  |  |  |  |  |  |  |
|  |  |  |  |  |  |  |  |  |  |  |  |  |  |  |
|  |  |  |  |  |  |  |  |  |  |  |  |  |  |  |
| Notch GoF | NICD=1 |  |  |  |  |  |  |  |  | Cell cycle arrest | FDCP | [3] | Metastasis stable state can be reached in particular conditions: only if all p53 family members are OFF. |  |
|  |  |  |  |  |  |  |  |  |  | Impaired differentiation | Mouse model | [4] |  |  |
|  |  |  |  |  |  |  |  |  |  |  |  |  |  |  |
| TGF-β GoF | TGFbeta=1 |  |  |  |  |  |  |  |  | metastasis | primary CRC in SCID mouse model | [5] | When apoptosis or HS are observed in the stable states, growth factors are OFF. It is a particular condition met in the centre of the tumour. |  |
|  |  |  |  |  |  |  |  |  |  |  |  |  |  |  |
|  |  |  |  |  |  |  |  |  |  | EMT (metastasis cannot be shown because of cell lines) | MCF10A; immortalised mouse lung fibroblast | [6] |  |  |
| APC LoF  Notch GoF | CTNNB1=1  NICD=1 |  |  |  |  |  |  |  |  | In Notch^+^APC^+/-^ mouse more adenomas than in APC^+/-^. Differentiation into goblet cells | Mouse model | [7] | same phenotypes as CTNNB1 GoF |  |
|  |  |  |  |  |  |  |  |  |  |  |  |  |  |  |
| APC LoF  Notch LoF | CTNNB1=1  NICD=0 |  |  |  |  |  |  |  |  | No difference in tumorigenesis compared to APC^+/-^. Inhibition of differentiation | Mouse model | [8] | same phenotypes as CTNNB1 GoF |  |
|  |  |  |  |  |  |  |  |  |  |  |  |  |  |  |
| APC LoF  KRAS GoF | CTNNB1=1  ERK=1 |  |  |  |  |  |  |  |  | adenoma, invasive carcinoma, increased proliferation | Mouse model | [9] | same as CTNNB1 GoF. Differentiation is not a phenotype of the model |  |
|  |  |  |  |  |  |  |  |  |  |  |  |  |  |  |

| Mutation | Model variables | Apoptosis | CCA | EMT | Invasion | Migration | Metastasis | HS |  | Experimental results | Experimental model | Ref. | Comments for model results | limitations |
| --- | --- | --- | --- | --- | --- | --- | --- | --- | --- | --- | --- | --- | --- | --- |
| KRAS GoF | ERK=1 |  |  |  |  |  |  |  |  | Observed adenomas and invasive adenocarcinomas, no distant metastasis; only activation of ERK1/2 but not AKT. | Mouse model | [10] | Model shows adenoma as phenotype but also metastasis under certain conditions: inactive p53 with active NICD and AKT2 |  |
|  |  |  |  |  |  |  |  |  |  |  |  |  |  |  |
|  |  |  |  |  |  |  |  |  |  | In [11]active AKT is observed during early time points. Lung adenoma and adenocarcinoma | Mouse model | [11] |  |  |
|  |  |  |  |  |  |  |  |  |  | Increased wound healing and number of colonies with soft agar assay. These assays shows the increased ability of metastasis of KRAS mutants | DLD1, Hec1A, HMEC | [12] |  |  |
| KRAS GoF  Notch GoF | ERK=1  NICD=1 |  |  |  |  |  |  |  |  | Metaplasia | Mouse model | [13] | Absence of p53 family members allows metastasis to occur in the last case |  |
|  |  |  |  |  |  |  |  |  |  | Adenoma, adenocarcinoma G4, G5  observed migration and invasion | Mouse model | [14] |  |  |
|  |  |  |  |  |  |  |  |  |  |  |  |  |  |  |
| HRAS GoF | ERK=1 |  |  |  |  |  |  |  |  | EMT, migration, increased snai2, vim and cdh2 expression; cdh1 and CTNNB1 down-regulated in mice that show cancer | NIH3T3 MCF10A | [15] | For the stable states with CDH1 down and CTNNB1 down, EMT or metastasis are obtained and not cell cycle arrest and not HS | Metastasis cannot be observed in cell lines |
|  |  |  |  |  |  |  |  |  |  |  |  |  |  |  |
|  |  |  |  |  |  |  |  |  |  |  |  |  |  |  |
|  |  |  |  |  |  |  |  |  |  |  |  |  |  |  |
| Snai2 GoF | Snai2=1 |  |  |  |  |  |  |  |  | EMT; migration; vim UP; cdh1 and CTNNB1 down. | NIH3T3 MCF10A | [15] | The stable state HS does not correspond to CDH1 ON, but corresponds to absence of AKT2. The stable states are with either EMT or metastasis. Metastasis occurs when p63 is OFF. If p63 is present, invasion and migration are inhibited |  |
|  |  |  |  |  |  |  |  |  |  |  |  |  |  |  |
|  |  |  |  |  |  |  |  |  |  |  |  |  |  |  |
|  |  |  |  |  |  |  |  |  |  |  |  |  |  |  |
| Twists1 GoF | Twist1=1 |  |  |  |  |  |  |  |  | EMT, vim and cdh2 UP | MCF7  HELA | [16] | There are stable states for which vim is OFF but they also lead to EMT. For these states, p53 is ON and AKT2 is OFF |  |
|  |  |  |  |  |  |  |  |  |  |  |  |  |  |  |

| Mutation | Model variables | Apoptosis | CCA | EMT | Invasion | Migration | Metastasis | HS |  | Experimental results | Experimental model | Ref. | Comments for model results | limitations |
| --- | --- | --- | --- | --- | --- | --- | --- | --- | --- | --- | --- | --- | --- | --- |
| p53 LoF | p53=0 |  |  |  |  |  |  |  |  | normal development, no tumours observed | Mouse model | [17] | Our mathematical model showed AKT2 and ERK are active with stable state leading to metastasis and EMT due to activation of Notch. Not for the apoptotic or HS stable states |  |
|  |  |  |  |  |  |  |  |  |  |  |  |  |  |  |
|  |  |  |  |  |  |  |  |  |  |  |  |  |  |  |
|  |  |  |  |  |  |  |  |  |  |  |  |  |  |  |
| TGF-β ectopic expression in p53 LoF | TGFbeta=1  p53=0 |  |  |  |  |  |  |  |  | EMT faster than with TGFbeta alone | NMuMG | [18] | No investigation of neither invasion nor migration only at the EMT regulators.  No conclusion about speed can be made with our formalism |  |
|  |  |  |  |  |  |  |  |  |  |  |  |  |  |  |
|  |  |  |  |  |  |  |  |  |  |  |  |  |  |  |
| U0126-induced MEK inhibition and TGF-β ectopic administration | TGFbeta=1  ERK=0 |  |  |  |  |  |  |  |  | Observed cell death. Vimentin is still present; no other information about EMT markers is available. No migration | H1666  H322  H358 | [19] | Invasion but no migration. In our model invasion happens when MMPs are secreted that dissolve the basal membrane |  |
|  |  |  |  |  |  |  |  |  |  |  |  |  |  |  |
|  |  |  |  |  |  |  |  |  |  |  |  |  |  |  |
| AKT2 GoF | AKT2=1 |  |  |  |  |  |  |  |  | migration and metastasis | CaOV3, SK-OV-3, OV2008, MDA-MB-435, MDA-MB-231, T47D, MCF7, HBL-100, SKBR3, PC3,PANC-1 | [20] | In the stable state where only EMT occurs, TGFbeta pathway is not activated |  |
|  |  |  |  |  |  |  |  |  |  |  |  |  |  |  |
| Notch GoF  p53 LoF | NICD=1  p53=0 |  |  |  |  |  |  |  |  | Mouse model | Mouse model | [21] |  |  |
| APC LoF  KRAS GoF  p53 LoF | CTNNB1=1  ERK=1  p53=0 |  |  |  |  |  |  |  |  | Not yet performed |  |  | Mutation sequence for CRC proposed by Vogelstein [22] |  |
| Notch GoF  p53 LoF  TGF-β LoF | NICD=1  p53=0  TGFbeta=0 |  |  |  |  |  |  |  |  | Not yet performed |  |  | Metastasis is suppressed |  |

| Mutation | Model variables | Apoptosis | CCA | EMT | Invasion | Migration | Metastasis | HS |  | Experimental results | Experimental model | Ref. | Comments for model results | limitations |
| --- | --- | --- | --- | --- | --- | --- | --- | --- | --- | --- | --- | --- | --- | --- |
| KRAS GoF  PTEN LoF | ERK=1  AKT1=1  AKT2=1 |  |  |  |  |  |  |  |  | macrometastasis | Mouse model | [23] | Cdh1 is not ON in the HS |  |
|  |  |  |  |  |  |  |  |  |  |  |  |  |  |  |
|  |  |  |  |  |  |  |  |  |  |  |  |  |  |  |
| PTEN LoF | AKT1=1  AKT2=1 |  |  |  |  |  |  |  |  | adenomacarcinoma; p53 and p63 are down-regulated | Mouse model | [23] | p53 family members are always OFF. However, metastasis cannot be observed because of AKT2 presence. Our assumption in the model is that AKT1 is inhibiting migration. Cdh1 is not ON in the HS |  |
|  |  |  |  |  |  |  |  |  |  |  |  |  |  |  |
|  |  |  |  |  |  |  |  |  |  | invasive adenocarcinoma, later metastasis | Mouse model | [24] |  |  |
| p53^+/+^ PTEN^-/-^ Apc^-/-^ | p53=1  AKT1=1  AKT2=1  CTNNB1=1 |  |  |  |  |  |  |  |  | metastasis (liver) all tumours | Mouse model | [25] | Migration is inhibited because of presence of AKT1 |  |
| p53^-/-^ PTEN^-/-^ Apc^-/-^ | p53=0  AKT1=1  AKT2=1  CTNNB1=1 |  |  |  |  |  |  |  |  | one case of distant metastasis (lung) | Mouse model | [25] | Migration is inhibited because of presence of AKT1 |  |

CCA: Cell Cycle Arrest; HS: homeostatic state; Ref.: references. Comments in blue colour mean that the experiments were performed in a mouse model. Comments in magenta colour indicate that the experiments have been conducted in cell lines. Black dots mean that node’s activity is 1.

References:

1. Fodde R, Edelmann W, Yang K, van Leeuwen C, Carlson C, et al. (1994) A targeted chain-termination mutation in the mouse Apc gene results in multiple intestinal tumors. Proc Natl Acad Sci U S A 91: 8969–8973.

2. Derksen PWB, Liu X, Saridin F, van der Gulden H, Zevenhoven J, et al. (2006) Somatic inactivation of E-cadherin and p53 in mice leads to metastatic lobular mammary carcinoma through induction of anoikis resistance and angiogenesis. Cancer Cell 10: 437–449. doi:10.1016/j.ccr.2006.09.013.

3. Henning K, Heering J, Schwanbeck R, Schroeder T, Helmbold H, et al. (2008) Notch1 activation reduces proliferation in the multipotent hematopoietic progenitor cell line FDCP-mix through a p53-dependent pathway but Notch1 effects on myeloid and erythroid differentiation are independent of p53. Cell Death Differ 15: 398–407. doi:10.1038/sj.cdd.4402277.

4. Fre S, Huyghe M, Mourikis P, Robine S, Louvard D, et al. (2005) Notch signals control the fate of immature progenitor cells in the intestine. Nature 435: 964–968. doi:10.1038/nature03589.

5. Calon A, Espinet E, Palomo-Ponce S, Tauriello DVF, Iglesias M, et al. (2012) Dependency of colorectal cancer on a TGF-β-driven program in stromal cells for metastasis initiation. Cancer Cell 22: 571–584. doi:10.1016/j.ccr.2012.08.013.

6. Iliopoulos D, Polytarchou C, Hatziapostolou M, Kottakis F, Maroulakou IG, et al. (2009) MicroRNAs differentially regulated by Akt isoforms control EMT and stem cell renewal in cancer cells. Sci Signal 2: ra62. doi:10.1126/scisignal.2000356.

7. Fre S, Pallavi SK, Huyghe M, Laé M, Janssen K-P, et al. (2009) Notch and Wnt signals cooperatively control cell proliferation and tumorigenesis in the intestine. Proc Natl Acad Sci U S A 106: 6309–6314. doi:10.1073/pnas.0900427106.

8. Peignon G, Durand A, Cacheux W, Ayrault O, Terris B, et al. (2011) Complex interplay between β-catenin signalling and Notch effectors in intestinal tumorigenesis. Gut 60: 166–176. doi:10.1136/gut.2009.204719.

9. Janssen K-P, Alberici P, Fsihi H, Gaspar C, Breukel C, et al. (2006) APC and oncogenic KRAS are synergistic in enhancing Wnt signaling in intestinal tumor formation and progression. Gastroenterology 131: 1096–1109. doi:10.1053/j.gastro.2006.08.011.

10. Janssen K-P, El-Marjou F, Pinto D, Sastre X, Rouillard D, et al. (2002) Targeted expression of oncogenic K-ras in intestinal epithelium causes spontaneous tumorigenesis in mice. Gastroenterology 123: 492–504.

11. Guerra C, Mijimolle N, Dhawahir A, Dubus P, Barradas M, et al. (2003) Tumor induction by an endogenous K-ras oncogene is highly dependent on cellular context. Cancer Cell 4: 111–120.

12. Vartanian S, Bentley C, Brauer MJ, Li L, Shirasawa S, et al. (2013) Identification of mutant K-Ras-dependent phenotypes using a panel of isogenic cell lines. J Biol Chem 288: 2403–2413. doi:10.1074/jbc.M112.394130.

13. De La O J-P, Emerson LL, Goodman JL, Froebe SC, Illum BE, et al. (2008) Notch and Kras reprogram pancreatic acinar cells to ductal intraepithelial neoplasia. Proc Natl Acad Sci U S A 105: 18907–18912. doi:10.1073/pnas.0810111105.

14. Baumgart A, Mazur PK, Anton M, Rudelius M, Schwamborn K, et al. (2014) Opposing role of Notch1 and Notch2 in a Kras(G12D)-driven murine non-small cell lung cancer model. Oncogene. doi:10.1038/onc.2013.592.

15. Vuoriluoto K, Haugen H, Kiviluoto S, Mpindi J-P, Nevo J, et al. (2011) Vimentin regulates EMT induction by Slug and oncogenic H-Ras and migration by governing Axl expression in breast cancer. Oncogene 30: 1436–1448. doi:10.1038/onc.2010.509.

16. Li J, Zhou BP (2011) Activation of β-catenin and Akt pathways by Twist are critical for the maintenance of EMT associated cancer stem cell-like characters. BMC Cancer 11: 49. doi:10.1186/1471-2407-11-49.

17. Jonkers J, Meuwissen R, van der Gulden H, Peterse H, van der Valk M, et al. (2001) Synergistic tumor suppressor activity of BRCA2 and p53 in a conditional mouse model for breast cancer. Nat Genet 29: 418–425. doi:10.1038/ng747.

18. Termén S, Tan E-J, Heldin C-H, Moustakas A (2013) p53 regulates epithelial-mesenchymal transition induced by transforming growth factor β. J Cell Physiol 228: 801–813. doi:10.1002/jcp.24229.

19. Buonato JM, Lazzara MJ (2014) ERK1/2 blockade prevents epithelial-mesenchymal transition in lung cancer cells and promotes their sensitivity to EGFR inhibition. Cancer Res 74: 309–319. doi:10.1158/0008-5472.CAN-12-4721.

20. Arboleda MJ, Lyons JF, Kabbinavar FF, Bray MR, Snow BE, et al. (2003) Overexpression of AKT2/protein kinase Bbeta leads to up-regulation of beta1 integrins, increased invasion, and metastasis of human breast and ovarian cancer cells. Cancer Res 63: 196–206.

21. Chanrion M, Kuperstein I, Barrière C, El Marjou F, Cohen D, et al. (2014) Concomitant Notch activation and p53 deletion trigger epithelial-to-mesenchymal transition and metastasis in mouse gut. Nat Commun 5: 5005. doi:10.1038/ncomms6005.

22. Morán A, Ortega P, de Juan C, Fernández-Marcelo T, Frías C, et al. (2010) Differential colorectal carcinogenesis: Molecular basis and clinical relevance. World J Gastrointest Oncol 2: 151–158. doi:10.4251/wjgo.v2.i3.151.

23. Mulholland DJ, Kobayashi N, Ruscetti M, Zhi A, Tran LM, et al. (2012) Pten loss and RAS/MAPK activation cooperate to promote EMT and metastasis initiated from prostate cancer stem/progenitor cells. Cancer Res 72: 1878–1889. doi:10.1158/0008-5472.CAN-11-3132.

24. Wang S, Gao J, Lei Q, Rozengurt N, Pritchard C, et al. (2003) Prostate-specific deletion of the murine Pten tumor suppressor gene leads to metastatic prostate cancer. Cancer Cell 4: 209–221.

25. Wu R, Baker SJ, Hu TC, Norman KM, Fearon ER, et al. (2013) Type I to type II ovarian carcinoma progression: mutant Trp53 or Pik3ca confers a more aggressive tumor phenotype in a mouse model of ovarian cancer. Am J Pathol 182: 1391–1399. doi:10.1016/j.ajpath.2012.12.031.
